# Maternal experiences of ethnic discrimination and subsequent birth outcomes in Aotearoa New Zealand

**DOI:** 10.1186/s12889-019-7598-z

**Published:** 2019-09-18

**Authors:** Zaneta Thayer, Laia Bécares, Polly Atatoa Carr

**Affiliations:** 1 Department of Anthropology & Ecology, Evolution, Environment and Society Program, Dartmouth College, Hanover, New Hampshire USA; 2 Applied Social Science, University of Sussex, Brighton, UK; 3 National Institute of Demographic and Economic Analysis, University of Waikato, Hamilton, New Zealand

**Keywords:** Racism, Health disparities, Indigenous health, Intergenerational effects

## Abstract

**Background:**

Interpersonal discrimination experience has been associated with adverse birth outcomes. Limited research has evaluated this relationship within multicultural contexts outside the United States where the nature and salience of discrimination experiences may differ. Such research is important in order to help identify protective and risk factors that may mediate the relationship between discrimination experience and adverse birth outcomes.

**Methods:**

Evaluated the relationship between perceived discrimination, as measured in pregnancy, with birth weight and gestation length among Māori, Pacific, and Asian women from Aotearoa New Zealand (*N* = 1653).

**Results:**

Thirty percent of the sample reported some type of unfair treatment that they attributed to their ethnicity. For Māori women specifically, unfair treatment at work (β = − 243 g) and in acquiring housing (β = − 146 g) were associated with lower birth weight when compared to Māori women not experiencing these types of discrimination, while an ethnically motivated physical attack (β = − 1.06 week), and unfair treatment in the workplace (β = − 0.95 week), in the criminal justice system (β = − 0.55 week), or in banking (β = − 0.73 week) were associated with significantly shorter gestation.

**Conclusions:**

Despite a high prevalence of discrimination experience among women from all ethnic groups, discrimination experience was a strong predictor of lower birth weight and shorter gestation length among indigenous Māori women only. Additional research is needed to better understand the risk and protective factors that may moderate the relationship between discrimination experience and adverse birth outcomes among women from different ethnic groups.

## Background

Aotearoa New Zealand, like many other developed countries, has substantial inequities in health, with lower rates of morbidity and mortality among white New Zealanders (New Zealand European), compared particularly to those of indigenous (Māori) ethnicity [1, 2]. One of the pathways through which health inequities can emerge is through differential exposure to psychosocial stressors, such as discrimination [3]. Discrimination can be both interpersonal and institutional [4]. Studies in Aotearoa New Zealand and elsewhere, including the United States and the United Kingdom, have found that reported experiences of interpersonal discrimination are associated with outcomes such as elevated blood pressure, altered stress physiology functioning, altered health behaviors, and increased risk of depression [5, 6]. Discrimination is thought to lead to poor health via several mechanisms. First, it increases exposure to adverse social circumstances, including insecure employment, low income, unsafe housing and neighborhood conditions, targeted marketing of harmful products, and suboptimal provision of social welfare [7]. Second, discrimination is also associated with poor health care access and quality of care [8–10]. Third, internalizing negative messages may lead to decreased self-esteem and poorer mental health [4]. Fourth, exposure to stressors, such as interpersonal discriminatory treatment, may result in physiological changes and to the subsequent onset and worsening of disease [3, 11, 12].

In addition to experiences of discrimination shaping individual health, there is increasing evidence that experiencing discrimination in prior generations can impact biology and health in subsequent generations [13–23]. For example, maternal experiences of ethnic discrimination have been associated with the development of adverse birth outcomes, including low birth weight and preterm birth, in studies from the United States [13–15, 21]. Notably, lower birth weight and preterm birth predict not only short term disability, but chronic disease risk in later life as well [24–27]. It is therefore possible that well documented inequities in chronic disease documented among ethnic minorities in adulthood may trace back, in part, to maternal experience of prenatal stress, including experiences of ethnic discrimination [28].

Maternal experiences of discrimination may influence fetal development and timing of parturition through impacts on stress physiology functioning. For example, we have previously reported that the stress hormone cortisol is higher in pregnant women reporting discrimination experience in Aotearoa New Zealand [20]. Maternal cortisol has been separately associated with fetal growth rate, birth size, and gestation length [29]. Reports of ethnic discrimination are high among New Zealand women [30], and substantial inequities in birth outcomes exist across women of different ethnicities [31]. Further, self-reported experience of ethnic discrimination in adulthood in Aotearoa New Zealand has been shown to be significantly associated with reduced self-rated health; lower physical health and functioning; lower mental health; health risk (including tobacco smoking); and cardiovascular disease [32, 33]. Prior research in Aotearoa New Zealand has not, however, evaluated whether birth outcomes are associated with these maternal experiences of discrimination.

Given this background, the purpose of this paper is to understand whether maternal experience of ethnic discrimination predicts birth outcomes among a large and ethnically diverse cohort of women in Aotearoa New Zealand, where measures were collected longitudinally – both before and after birth. The results of this analysis are important for understanding whether the previously documented relationship between interpersonal discrimination experience and adverse birth outcomes may differ based on cultural context of those experiences.

## Methods

Data for this paper come from the Growing Up in New Zealand (GUINZ) longitudinal cohort study. Using multiple strategies, pregnant women were invited to participate in GUINZ if they had an estimated birth date between 25 April 2009 and 25 March 2010, and were living within a geographic area defined by the Auckland, Counties-Manukau, or Waikato District Health Board regions in the North Island of Aotearoa New Zealand [34]. The recruited pregnant women (*n* = 6822) and the resulting main cohort of their 6853 children are generally comparable to New Zealand national birth statistics in relation to maternal age, ethnicity, parity and indicators of socioeconomic position [35]. The sample represents approximately 11% of all births in Aotearoa New Zealand during the study period. Data for the present analyses are derived from data collected over two time points: antenatally and at 6 weeks postnatal. Ethical approval was obtained from the Ministry of Health Northern Y Regional Ethics Committee (NTY/08106/055). Written informed consent for interviews and data linkage was completed by each participant. As described below, our analyses examined the association between experiences of ethnic discrimination (independent variable) and birth outcomes (dependent variable), while adjusting for several covariates.

### Ethnic discrimination

In the antenatal questionnaire (Additional file 1), women were asked a series of questions regarding lifetime and past year experiences of ethnic discrimination [34]. This included physical attacks and verbal attacks that individuals attributed to their ethnicity. In addition, participants were asked if they had ever felt they had been treated unfairly because of their ethnicity across a range of domains. This included by a health professional; in employment settings; in the housing market; by the police, the justice system, or the corrections department; by the banking system (asking for loans, a mortgage, hire purchase or credit cards); and when attending a place of learning. For all questions, participants were able to answer ‘yes, within the past 12 months;’ ‘Yes, more than 12 months ago;’ and ‘No.’ We analyzed whether individuals had ever experienced each of the attack or unfair treatment variables (yes/no).

### Birth outcomes

Women gave consent for linkage to the birth information collected by maternity hospitals and District Health Boards. Gestation length and birth weight were analyzed as continuous variables, with the latter adjusted for gestational age.

### Ethnicity

In the antenatal questionnaire, women were asked “which ethnic group or groups do you belong to?” Those women that identified more than one ethnic group [36] were then asked to self-prioritize their ethnicity (“which is your main ethnic group; that is, the one you identify with most?”). Ethnic identification responses to these questions were aggregated for the purposes of these analyses into five broad groups: European, Māori, Pacific, Asian and Other categories. Given the focus on understanding how discrimination predicted health inequities, analyses were restricted to participants of Māori, Pacific, and Asian ethnic identification (*N* = 2828).

### Covariates

Maternal age in pregnancy (years), maternal body mass index (BMI) (kilograms/meter^2^), yearly household income (1 = ≤ 20,000 New Zealand Dollars (NZD); 2 = 20,000–29,999NZD; 3 = 30,000–49,999NZD; 4 = 50,000–69,999NZD; 5 = 70,000–99,999NZD; 6 = 100,000–149,999NZD; 7 ≥ 150,000NZD), maternal education (0 = Trade certification/National Certificate Levels 1–4; 1 = Diploma below bachelors or National Certificate 5 or 6; 2 = Bachelors Degree; 3 = Bachelors degree with honors or postgraduate diploma; 4 = Masters degree; 5 = PhD), mother’s relationship status in pregnancy (with or without partner), and smoking in pregnancy (yes/no) were assessed during the antenatal questionnaire and were included as covariates in our multivariate regression models. Offspring sex (male/female) was collected at the 6 week questionnaire and was also included as a covariate in multivariate models. Women with incomplete data for any covariates were excluded from the analysis, resulting in a final sample size of 1653.

### Analysis

We first performed a univariate analysis to describe sample characteristics, both within the entire sample and stratified by ethnicity. We next evaluated bivariate associations between all study variables by calculating Pearson correlation coefficients. We then used multivariate regression to evaluate the relationship between each of the discrimination variables and birth weight and gestation length, respectively, while adjusting for covariates. Multivariate models were run separately for each of the three ethnic groups.

## Results

Summary statistics are provided in Table 1. Pacific women gave birth to infants with the highest birth weight (mean = 3627 g (g), standard deviation (SD) = 595 g), while Asian women gave birth to infants with the lowest (mean = 3242 g, SD = 542 g). Twenty-six percent of the sample reported a verbal attack, with Māori reporting a high of 33%. Four percent of the sample reported experiencing a physical attack. Thirty percent of the sample reported having experienced at least one type of unfair treatment, with Māori women reporting the most at 37%.

**Table 1:** Summary statistics of study sample

	Total sample (*N* = 1653)	Māori (*N* = 510)	Pacific (*N* = 452)	Asian (*N* = 691)
Age (years)	29.5 (5.6)	28.7 (6.2)	29.5 (6.2)	30.2 (4.7)
Education category	2.07 (1.10)	1.8 (1.05)	1.7 (0.98)	2.6 (1.0)
Household income category	4.2 (1.6)	4.3 (1.6)	3.9 (1.6)	4.3 (1.5)
Had partner/married in pregnancy	96% (1594)	93% (473)	95% (430)	100% (691)
Smoked in pregnancy	23% (373)	38% (193)	32% (143)	5% (37)
BMI (kg/m^2^)	26.3 (6.8)	27.6 (6.2)	31.0 (7.6)	22.3 (3.7)
Birth weight (g)	3481 (584)	3475 (568)	3627 (595)	3242 (542)
Low birth weight (< 2500 g)	5.0% (340)	5.0% (47)	3.0% (30)	6.5% (65)
Gestation length (weeks)	39.1 (1.9)	39.1 (1.8)	39.1 (1.9)	38.9 (1.8)
Preterm birth (< 37 weeks)	6.4% (436)	5.8% (55)	5.4% (54)	6.4% (64)
Ever experienced verbal attack	26% (427)	33% (170)	20% (90)	24% (167)
Ever experienced physical attack	4% (59)	5% (25)	4% (17)	2% (17)
Any unfair treatment	30% (498)	37% (189)	28% (127)	27% (182)

Mean and standard deviation are provided for continuous variables, while percentage and total number are presented for categorical variables

Bivariate analyses of the entire sample (Table 2) demonstrated that higher maternal age was positively associated with education, income, and BMI, and negatively associated with gestation length, smoking, and being single in pregnancy. Household income was higher among those with more education, and was associated with lower BMI and not smoking in pregnancy. Birth weight was strongly associated with maternal BMI.

**Table 2:** Pearson’s correlation coefficient (r) for study variables

	1	2	3	4	5	6	7	8	9	10
1. Age	–									
2. Education	0.22*									
3. Household income	0.23*	0.24*								
4. Have partner/married	0.06*	0.14*	0.13*							
5. Smoking in pregnancy	−0.18*	−0.29*	−0.16*	−0.23*						
6. BMI	0.06*	− 0.19*	− 0.06*	− 0.05†	0.17*					
7. Birth weight (g)	−0.0003	− 0.03	0.03	− 0.02	0.04†	0.25*				
8. Gestation length	−0.05*	0.03	0.07*	−0.001	−0.04	0.01	0.56*			
9. Verbal attack	0.05†	0.11*	0.12*	−0.02	−0.005	−0.05†	0.008	0.03		
10. Physical attack	0.02	0.03	−0.02	−0.05*	0.03	0.04†	0.002	−0.05*	0.09*	
11. Any unfair treatment	−0.005	0.06*	0.02	−0.06*	0.06*	0.006	0.02	−0.002	0.37*	0.13*

* = *P* < 0.05; † = *P* < 0.10

Women who had more education and higher income were significantly more likely to report having experienced a verbal attack, and higher education was associated with being significantly more likely to report any unfair treatment. Physical attack in pregnancy was associated with significantly shorter gestation length in the entire sample.

**Fig. 1:**
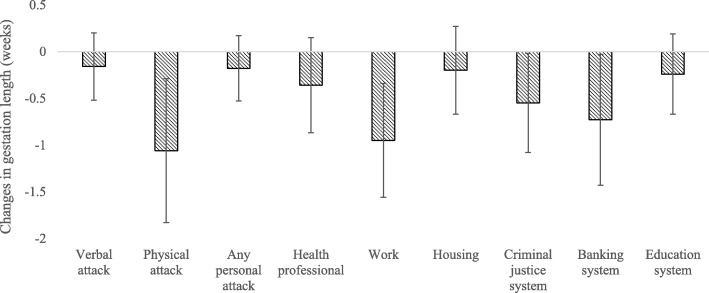
There was a general trend for lower birth weight in response to discrimination experience among Māori mothers. Figure reports Beta coefficients and 95% CI

Table 3 presents the relationships between discrimination experience measures and birthweight for women from the different ethnic categories after adjusting for covariates. There was an overall trend for Māori women reporting ethnic discrimination experience to have lower birth weight infants relative to Māori women not reporting discrimination (Fig. 1). Specifically, Māori women who experienced unfair treatment at work (β = − 243 g 95% CI − 425 g, − 60.2 g) and in acquiring housing (β = − 146 g, 95% CI − 286 g, − 6 g) were more likely to have an infant with lower birth weight compared to Māori women who did not report experiences of discrimination. Conversely, Asian women who reported housing discrimination had infants with higher birth weight (β = 188 g, 95% CI 7 g, 369), when compared to Asian women who didn’t report experiencing ethnic discrimination. There was no relationship between any of the discrimination variables and birth weight for Pacific women.

**Table 3:** Associations between lifetime experiences of ethnic discrimination and birth weight (grams) among Māori, Pacific, and Asian women

	Māori β coeff (95% CI)	Pacific β coeff (95% CI)	Asian β coeff (95% CI)
Personal attack			
Verbal attack	− 77.2 (− 184, 30.5)	21.8 (− 116, 160)	83.0 (− 11.3, 177)
Physical attack	− 190 (− 423, 42.9)	158 (− 124, 442)	− 3.08 (− 258, 252)
Any personal attack	−84.7 (− 190, 20.9)	24.8 (− 109, 159)	75.7 (− 16.1, 167)
Unfair treatment			
Health professional	−13.0 (− 166, 140)	142 (− 46.7, 330)	4.98 (− 194, 204)
Work	**−243 (− 425, − 60.2)**	137 (− 46.7, 321)	49.2 (− 66.0, 164)
Housing	**−146 (−286, −5.93)**	15.1 (− 177, 207)	**188 (7.04, 369)**
Criminal justice system	−95.8 (− 255, 64.0)	138 (−91.6, 369)	2.12 (− 295, 299)
Banking system	− 122 (− 333, 89.7)	246 (−34.4, 527)	119 (− 314, 553)
Education system	−63.9 (− 193, 65.7)	79.9 (− 108, 268)	−86.6 (− 239, 66.3)

All models adjusted for maternal age, education, relationship status, smoking, BMI, offspring sex, and household income; Bold = *P* < 0.05

**Fig. 2:**
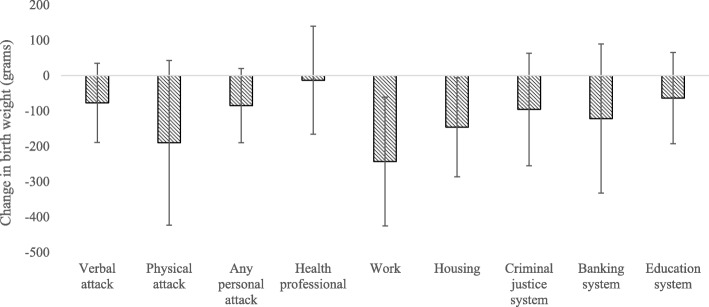
There was a general trend for shorter gestation length in response to discrimination experience among Māori mothers. Figure reports Beta coefficients and 95% CI

The relationships between ethnic discrimination measures and gestation length after adjusting for covariates are presented in Table 3. There was an overall trend for Māori women reporting ethnic discrimination experience to have shorter gestation length relative to Māori women not reporting discrimination (Fig. 2). Māori women who reported an ethnically motivated physical attack (β = − 1.06 week, 95% CI − 1.8 week, 0.3 week) or who experienced unfair treatment in the workplace (β = − 0.95 week, 95% CI = − 1.6 week, 0.3 week), in the criminal justice system (β = − 0.55 week, 95% CI = − 1.1 week, 0.02 week), or in banking (β = − 0.73 week, 95% CI = − 1.4 week, 0.02 week), had shorter gestation length. There were no significant associations between ethnic discrimination measures and gestation length for Pacific or Asian women (Table 4).

**Table 4:** Associations between lifetime experiences of ethnic discrimination and gestation length (weeks) among Māori, Pacific, and Asian women

	Māori β coeff (95% CI)	Pacific β coeff (95% CI)	Asian β coeff (95% CI)
Personal attack			
Verbal attack	−0.16 (− 0.52, 0.19)	0.33 (− 0.91, 0.76)	0.18 (− 0.10, 0.48)
Physical attack	**−1.06 (− 1.83, − 0.28)**	0.35 (− 0.52, 1.22)	−0.39 (− 1.19, 0.40)
Any personal attack	− 0.18 (− 0.53, 0.16)	0.29 (− 0.12, 0.70)	0.13 (− 0.15, 0.42)
Unfair treatment			
Health professional	− 0.36 (− 0.87, 0.15)	0.25 (− 0.32, 0.84)	−0.26 (− 0.88, 0.36)
Work	**− 0.95 (− 1.56, − 0.34)**	−0.40 (− 0.60, 0.52)	0.18 (− 0.17, 0.55)
Housing	−0.20 (− 0.67, 0.26)	0.09 (− 0.50, 0.69)	0.30 (− 0.26, 0.87)
Criminal justice system	**−0.55 (− 1.08, − 0.02)**	0.51 (− 0.19, 1.22)	0.45 (− 0.47, 1.39)
Banking system	**−0.73 (− 1.43, − 0.02)**	0.64 (− 0.21, 1.51)	0.33 (− 1.02, 1.70)
Education system	−0.24 (− 0.67, 0.18)	0.18 (− 0.39, 0.76)	−0.31 (− 0.79, 0.16)

All models adjusted for maternal age, education, relationship status, smoking, BMI, offspring sex, and household income; Bold = *P* < 0.05

## Discussion

In this study we aimed to understand the association between maternal experience of ethnic discrimination and birth outcomes among a large and ethnically diverse cohort of women and their children in Aotearoa New Zealand. We find that a very high prevalence of women reported experiencing discrimination. Women with higher education and income were more likely to report having experienced verbal attacks. While there was an overall association between physical attack experience and shorter gestation weight, multivariate analyses stratified by ethnicity demonstrated that lower birth weight and shorter gestation length were associated with discrimination experience only among Māori women. Specifically, Māori women reporting discrimination in response to unfair treatment at work and in acquiring housing were more likely to have lower birth weight babies than Māori women who did not report experiencing these types of ethnic discrimination. In addition, Māori women reporting a physical attack or experiencing unfair treatment because of their ethnicity in the workplace, in the criminal justice system, or in banking all had significantly shorter gestation length than Māori women who did not report these experiences. The effect sizes were also relatively large, with unfair treatment at work predicting a 243 g reduction in birth weight, and physical attacks in pregnancy being associated with a week shorter gestation length among Māori women. These effects were independent of maternal socioeconomic status and individual health behaviors that impact birth weight, such as smoking. Interestingly, this relationship did not exist for women within the broad Pacific ethnic group, and among Asian women those reporting discrimination because of their ethnicity in the housing market actually had higher birth weight infants.

The finding of discrimination experience being associated with adverse birth outcomes among Māori women is consistent with prior research that has predominately been conducted among African Americans in the United States [37]. Consistent with our finding, other studies of ethnic discrimination and health in Aotearoa New Zealand also report that Māori health outcomes tend to be most strongly associated with discrimination experience [30, 38]. The finding that this association only exists among Māori, despite high prevalence of discrimination experience among other ethnic groups within this cultural context, requires further examination. It is possible that the type, severity, chronicity and nature of ethnic discrimination experience varies among groups, and this difference in salience contributes to differences in how these experiences are internalized. For example, Māori have experienced a history of colonization in Aotearoa New Zealand. Therefore discrimination experienced in their own lands may be different than perceived discrimination experienced by Pacific and Asian communities with a more recent history in Aotearoa New Zealand. The experience of discrimination as described by Māori may also have different influences on accessibility to societal resources and health determinants such as income, employment and security. In prior work in this sample we have found that Māori women reported more objective stress exposures (e.g. financial stress) and perceived stress relative to women who self-identify as European or Asian (Farewell et al. in review). In addition, it has been reported that Māori experience the greatest burden of implicit and explicit ethnic bias in the delivery of services, such as health care [39–43]. Importantly, these structurally-related discrimination experiences may not even be fully reflected in the perceived discrimination measurement used here but could still influence health outcomes [43], suggesting that there could be even greater unmeasured health impacts of discrimination.

The inverse relationship between housing discrimination and birth weight among Asian women was unexpected. Since women of Asian ethnicity had the lowest birth weights overall, higher birth weight within this group may reflect a less healthy birth weight. In addition, many studies find a j-shaped relationship between discrimination experience and health outcomes, with individuals who do not report having experienced discrimination actually having worse outcomes than those reporting moderate amounts of discrimination [14, 44, 45]. It is possible that this is the case for Asian, but not Māori women, in the current sample.

In addition to suggesting that the impacts of discrimination on birth outcomes may differ depending on ethnicity and cultural context, these results also suggest that discrimination may impact birth outcomes through pathways beyond the commonly described impacts on stress physiology. For example, increased birth weight associated with discrimination experience could reflect differences in diet or physical activity levels in pregnancy. These results highlight the need to evaluate the relationship between discrimination and birth outcomes in diverse cohorts in order to understand both how the context of discrimination experience affects internalization of these exposures, as well as the different physiological and behavioral pathways that mediate these effects.

Our analysis adjusted for several confounders, including smoking in pregnancy. Smoking is strongly associated with a reduction in fetal growth [46]. Smoking in pregnancy is highly prevalent in the present sample (23%), particularly among Māori women (38%). Importantly, smoking could be a coping mechanism for stressors [47], such as discrimination experience. For example, urban black and Hispanic women from the United States reporting high discrimination had higher odds or prenatal smoking than those women reporting only moderate discrimination [48]. Therefore adjusting for smoking provides a minimum estimate of the potential association between discrimination and birth outcomes we report here.

Despite the strengths of this study, which includes a large sample size and longitudinal study design, there are several limitations to consider. While the sample is ethnically representative of the New Zealand population, there is nevertheless a potential for bias with respect to women for whom all data are available for analysis. In particular, a large number of women were missing information for pre-pregnancy weight, and therefore were excluded from the analysis due to the inability to adjust for maternal size. Another potential limitation of this study is that there were no follow up questions that asked about how women perceived their discrimination experiences. While many women were able to describe and recognize having had a discriminatory experience, it would be informative to know their affective responses to such experiences, such as whether or not it elicited anger or made them feel upset. Differences in affective responses could affect internalization of these experiences [49]. Future work should not only assess these responses, but also evaluate the factors, individual, social, or material, that predict these responses.

## Conclusion

We found the expected relationship between discrimination experience and adverse birth outcomes, but only among Māori women. The results of this analysis suggest that ethnic discrimination can have important impacts on health for Māori, particularly since lower birth weight offspring and infants born with shorter gestational age are more likely to experience adverse health outcomes across the life course. Since this finding was not present for Pacific and Asian women it suggests that it is important to understand the cultural context within which discrimination experience is taking place, as there could be differences in additional risk or protective factors that moderate the relationship between discrimination and health. More research in multi-ethnic contexts is therefore needed to fully understand the pathways through which discrimination may impact health.

## Supplementary information

**Additional file 1.** Antenatal Questionnaire; Description: Questionnaire instrument.

## Data Availability

The data that support the findings of this study are available from the Growing Up in New Zealand study, but restrictions apply to the availability of these data, which were used under license for the current study, and so are not publicly available. Data are however available from the authors upon reasonable request and with permission of Growing Up in New Zealand.

## Abbreviations

BMI: Body mass index
g: Grams
GUINZ: Growing Up in New Zealand
NZD: New Zealand Dollars
SD: Standard deviation
