# Supplementary material for: Maternal experiences of ethnic discrimination and subsequent birth outcomes in Aotearoa New Zealand
Source: BMC Public Health. 2019 Sep 18;19:1271. doi: 10.1186/s12889-019-7598-z (PMC6751673; doi:10.1186/s12889-019-7598-z)
Supplement: Supplementary file 1 — Additional file 1. Antenatal Questionnaire; Description: Questionnaire instrument. [file 12889_2019_7598_MOESM1_ESM.pdf]

Antenatal Data Collection Wave: Main Cohort

Mother Questionnaire

Copyright

This questionnaire is the copyright of *Growing Up in New Zealand*. Please apply in writing to the Data Access Coordinator to gain permission(s) to use any questions, tables or other information contained in this document.

Growing Up in New Zealand
University of Auckland Tamaki Campus, Bldg 730.313
261 Morrin Road, Glen Innes, Auckland 1072
PO Box 18288, Auckland 1743
Phone: 0508 476 946
Email: contact@growingup.co.nz

www.growingup.co.nz

© Growing Up in New Zealand 2009

Antenatal questionnaire: Mother

Code: ___________________ Interview date: _________________

Confirm Code _ _ _ _ _ _ Check Digit _ _
Spec Instruction- Loop back if incorrect

Consent Forms if no then thank and close.

Interviewer Instruction: Enter Respondent's details below. If Respondent refuses or no response please type in **99**

Note: No fields can be left blank.

Name: ____

Phone: (Home) _______________ (Work) ________________ (Mobile) _______________________

Email: ____

What is your preferred method of contact?
1 = Home Phone
2 = Work phone
3 = Mobile Phone
4 = Email
5 = Other please specify
6 = None

Confidentiality

Your personal information will be kept in a separate secure location to the main questionnaire data.

Your answers are completely confidential. No personal information such as your name or address will be shared with any other individual or agency.

Remember that there are no right or wrong answers and your honesty is greatly appreciated.

© Growing Up in New Zealand 2009

Section A: Health & Well-Being

I would like to ask you some questions about you and your health. Most of these questions relate to this pregnancy. They include questions about your activity, diet and eating habits, your health, and your plans for the baby.

BG1. What is your date of birth? Interviewer Instruction:

Enter Date = 01 **GO TO BG1A**
DK/Ref = 99 **GO TO BG3**

BG1A. Enter Date of Birth e.g. 25/02/1981 (D) - - (M) - -(Y) - -- - **GO TO BG2**
[RANGE: YEARS>1949; < 01/01/1995]

BG2. So are you (computer inserted number) years old?

Yes = 1 **GO TO BG3**
No = 2 **GO TO BG1A**

If incorrect at BG2 re-ask BG1

BG3. Do you know how much you weighed when you were born?
Interviewer Instruction : Probe for best response

Yes = 1 **GO TO BG4**
Yes Approximately = 2 **GO TO BG4**
DK/Ref = 8 **GO TO BG5**

BG4. Please state your weight when you were born?
[RANGE: lb: 1-22; oz: 0-15; kg/g: >450; <9999; nb, 1kg = 1000g]

Specify pounds = 01
Specify kilograms/grams = 02
DK/Ref = 99 **GO TO BG5**

BG4A. Enter weight

Interviewer Instruction: Use double or triple digits as required. **Do not enter a "." decimal point.**

	Lbs		Ozs	OR		Kgs		Gms

BG5. When you were born were you more than 3 weeks early?

Yes = 1
No = 2
DK/Ref = 9

Thinking about this pregnancy now.

PRG1. When is your estimated due date?

Enter date = 01 **GO TO PRG1A**
DO NOT READ OUT DK/Ref = 99 **GO TO PRG2**

PRG1A. Enter Date e.g. 25/06/2009 (D) - - (M) - -(Y) - -- -
[RANGE: >01/04/2009; <31/01/2010] If day is unknown enter as 00

PRG2. When was your last menstrual period, that is the date your period started?
Interviewer Instruction: Enter as double digits If Respondent is not sure of exact date and only knows the month enter as e.g. 00 06 08

Enter Date = 01 **GO TO PRG2A**
Never had a period = 97 **GO TO PRG6**
DO NOT READ OUT DK/Ref = 99 **GO TO PRG3**

PRG2A. Enter Date e.g. 25/06/2009 (D) - - (M) - -(Y) - -- -
[RANGE: >01/01/2000; < interview date]

PRG3. Prior to this pregnancy were your periods regular?

Yes = 1
No = 2
DK/Ref = 9

PRG4. Approximately how long was your usual cycle? That is, how many days are there between two menstrual periods.

Interviewer Instruction: Enter as double digits [RANGE 10-40]

	days

DO NOT READ OUT DK/Ref = 99

PRG5. How old were you when you began your periods?
Interviewer Instruction: Enter as double digits [RANGE 5-20]

	years old

DO NOT READ OUT DK/Ref = 99

PRG6. Was this pregnancy planned?

Yes = 1 **GO TO PRG7**
No = 2 **GO TO PRG10**
DO NOT READ OUT DK/Ref = 9 **GO TO PRG10**

© Growing Up in New Zealand 2009

PRG7. Approximately how long did it take to get pregnant?

Specify years and months = 01 **GO TO PRG7A**
Specify months only = 02 **GO TO PRG7A**
DO NOT READ OUT DK/Ref = 99 **GO TO PRG8**

PRG7A. Interviewer Instruction: Enter digits as required; If less than 1 month enter as 01 months [RANGE: years & months: 01-20 years; 00-11 months; months only: 01-24]

	years		months
	OR		months

DO NOT READ OUT DK/Ref = 99

PRG8. Did you have any treatment to assist you with becoming pregnant?

Yes = 1
No = 2 **GO TO PRG10**
DO NOT READ OUT DK/Ref = 9 **GO TO PRG10**

SHOWCARD PRG9
PRG9. What treatment was given? Code all Mentions Multiple Response

Fertility awareness and weight loss = 1
Ovulation induction (with Clomiphene Citrate) in the community = 2
Ovulation induction in an infertility centre/hospital = 3
Tubal surgery = 4
IVF treatment = 5
Other (Please specify) = 6
DO NOT READ OUT DK/Ref = 9

PRG10. How many weeks pregnant were you when you became aware that you were pregnant?

Enter weeks = 01 **GO TO PRG10A**
DO NOT READ OUT DK/Ref = 99 **GO TO PRG11**

PRG10A. Interviewer Instruction: Enter as double digits; <1 week enter as 01. [RANGE: 01-40]

	weeks

SHOWCARD PRG11-12

Have you experienced any nausea or morning sickness during this pregnancy?
Interviewer note: if severity of nausea changed within a trimester code the most severe nausea experienced

	No nausea (=0)	Mild nausea (nausea only) (=1)	Moderate nausea (occasionally sick) (=2)	Severe nausea (Regularly sick, can't hold meals) (=3)	DK/Ref **DO NOT READ OUT** (=9)
**PRG11.** In the first 3 months of pregnancy, did you have…	☐	☐	☐	☐	
**PRG12.** From the first 3 months of pregnancy until now, have you had…	☐	☐	☐	☐	

Thinking about any previous pregnancies.
PRG13. Have you ever been pregnant before?

Yes = 1
No = 2 **GO TO PRG16**
DO NOT READ OUT DK/Ref = 9 **GO TO PRG16**

PRG14. How many pregnancies continued to more than 24 weeks?

None = 0 **GO TO PRG16**
One = 1
Two = 2
Three = 3
Four = 4
More than four = 5
DO NOT READ OUT DK/Ref = 9

PRG15. Did you attend any childbirth preparation classes in a previous pregnancy?

Yes = 1
No = 2
DO NOT READ OUT DK/Ref = 9

SHOWCARD PRG16

PRG16. Have you attended any childbirth preparation classes for this pregnancy?

Yes = 1
No, but intend to = 2
No and don't intend to = 3
DK/Ref = 9

Thinking about your health now and in the past

PRG17. Did you have a Family doctor or GP before you became pregnant?

Yes = 1
No = 2
DK/Ref = 9

PRG18. Have you seen any Family doctor or GP since you became pregnant?

Yes = 1
No = 2 **GO TO PRG20**
DK/Ref = 9 **GO TO PRG20**

PRG19. Is this the same Family doctor or GP as the one you saw before you became pregnant?

Yes = 1
No = 2
DK/Ref = 9

PRG20. Do you know who the Family doctor or GP will be for your baby after s/he is born?

Yes = 1
No = 2 **GO TO PRG22**
DK/Ref = 9 **GO TO PRG22**

PRG21. Is this the same Family doctor or GP as the one you saw before you became pregnant?

Yes = 1
No = 2
DK/Ref = 9

PRG22. Do you have a lead maternity caregiver (LMC)?

Yes = 1
No = 2 **GO TO PRG27**
DK/Ref = 9 **GO TO PRG27**

PRG23. Did you have a choice of midwife or other lead maternity caregiver (LMC) during this pregnancy?

Yes = 1
No = 2
DK/Ref = 9

PRG24. How long did it take you to find a lead maternity caregiver (LMC) from the time you began looking?
Interviewer Instruction: Enter as double digits
[RANGE: 0-13; code 0 if <1 week; code 13 if 13+]

Number of weeks	
DK/Ref	99

SHOWCARD PRG25,27

PRG25. What type of lead maternity caregiver (LMC) do you have?

Choose as many as apply
GP (Family doctor) = 1
Independent midwife = 2
Hospital midwife = 3
Obstetrician = 4
Shared care GP and midwife = 5
Other Please specify = 6
DK/Ref = 9 **GO TO PRG28**

PRG26. Was this type of LMC your first choice?

Yes =1 **GO TO PRG28**
No = 2
DK/Ref = 9

SHOWCARD PRG25,27

PRG27. If you had a choice, who would you most prefer as your primary carer in this pregnancy

GP (Family doctor) = 1
Independent midwife = 2
Hospital midwife = 3
Obstetrician = 4
Shared care GP and midwife = 5
Other Please specify = 6
DK/Ref = 9

PRG28. Did you consult any complementary or alternative practitioner or a traditional healer during your pregnancy?

Yes = 1
No = 2 **GO TO PRG30**
DK/Ref = 9 **GO TO PRG30**

SHOWCARD PRG29

PRG29. Please indicate all those you consulted:

Acupuncturist = 1
Chiropractor = 2
Osteopath = 3
Massage therapist = 4
Homeopath = 5
Naturopath = 6
Spiritual healer = 7
Herbalist = 8
Traditional Chinese medicine practitioner = 9
Maori healer = 10
Pacific healer = 11
Feldenkrais or Alexander technique practitioner = 12
Aromatherapist = 13
Colour therapist = 14
Other Please specify = 98
DK/Ref = 99

The next questions are about your plans for your baby

SHOWCARD PRG30

PRG30. How are you intending to feed your baby when they are first born?

Breast = 1
Bottle = 2 **GO TO PRG34**
Both breast and bottle = 3
Haven't decided = 4
DK/Ref = 9

SHOWCARD PRG31-32

PRG31. How long do you think is best to breast feed your baby?

Up to 6 weeks = 1
Up to 3 months = 2
Up to 6 months = 3
Longer than 6 months = 4
DK/Ref = 9

SHOWCARD PRG31-32

PRG32. Ideally, how long would you like to breast feed for?

Up to 6 weeks = 1
Up to 3 months = 2
Up to 6 months = 3
Longer than 6 months = 4
DK/Ref = 9

PRG33. Do you think the length of time you will breastfeed your baby will be limited by your need to return to work?

Interviewer Note: If length of time is not limited because respondent works from home, code "No".

Yes = 1
No = 2
Maybe = 3
DK/Ref = 9

SHOWCARD PRG34

PRG34. Have you decided yet if you will have your child fully immunised?

Yes I have decided I will have my child fully immunised = 1
Yes I have decided I will have my child partially (selectively) immunised = 2
Yes I have decided I will not have my child immunised = 3
No I have not decided yet = 4
DK/Ref = 9

PRG35. During this pregnancy have you received or been told any information that is encouraging you to immunise this child once s/he is born?
Interviewer note: Clarify that the information was received during this pregnancy.

Yes =1
No = 2 **GO TO PRG37**
DK/Ref = 9 **GO TO PRG37**

SHOWCARD PRG36,38

PRG36. Where did you get this information from? PROBE TO NO

Choose as many as apply
Family/whanau = 1
Friends = 2
GP (Family doctor) = 3
Midwife = 4
Obstetrician = 5
Dietician/nutritionist = 6
Alternative health practitioner = 7
Antenatal class = 8
The internet = 9
Radio = 10
TV = 11
Books, magazines, newspaper = 12
Other Please specify = 13
DK/Ref = 99

PRG37. During this pregnancy have you received or been told any information that is discouraging you to immunise this child once s/he is born?

Yes =1
No = 2 **GO TO ACT1A**
DK/Ref = 9 **GO TO ACT1A**

SHOWCARD PRG36,38

PRG38. Where did you get this information from? PROBE TO NO

Choose as many as apply
Family/whanau = 1
Friends = 2
GP (Family doctor) = 3
Midwife = 4
Obstetrician = 5
Dietician/nutritionist = 6
Alternative health practitioner = 7
Antenatal class = 8
The internet = 9
Radio = 10
TV = 11
Books, magazines, newspaper = 12
Other Please specify = 13
DK/Ref = 99

Questions about how active you are

Thinking about how active you are now, and how this compares with before you were pregnant.

I'm going to ask you some questions about the time you spend being physically active over an average 7 day period. Please answer even if you don't consider yourself a very active person. Think about the activities you usually do at work, at home, to get from place to place, or as sport, exercise, leisure or recreation time. Each activity should be for at least 10 minutes.

How many days would you have done the following kinds of exercise or activities, for longer than 10 minutes at a time?

Firstly, vigorous exercise – which makes your heart beat rapidly AND leaves you breathing hard e.g. jogging, fast cycling, aerobics, vigorous swimming, heavy lifting and digging.

Interviewer note: If respondent asks, clarify that exercise/activity at work is included.

ACT1A. Before you became pregnant, how many days per week (0 – 7) did you do vigorous activity?

IF = 0 **GO TO ACT2A**

ACT1B. How long on average did you do this type of activity on these days (<30 mins, 30-60 min, more than 60 min)?

ACT2A. During the first 3 months of pregnancy, how many days per week (0 – 7) did you do vigorous activity?

IF = 0 **GO TO ACT3A**

ACT2B. How long on average did you do this type of activity on these days (<30 mins, 30-60 min, more than 60 min)?

ACT3A. Since the first 3 months of pregnancy, how many days per week (0 – 7) did you do vigorous activity?

IF = 0 **GO TO ACT4A**

ACT3B. How long on average did you do this type of activity on these days (<30 mins, 30-60 min, more than 60 min)?

SHOWCARD ACT1B-6B

**Vigorous exercise**	**A. Average days per week circle ONE for each time period**									**B. Average length circle ONE for each**			
									DK/Ref	1	2	3	DK/Ref
**ACT1.** Pre-pregnancy	0	1	2	3	4	5	6	7	9	<30	30-60	>60	9
**ACT2.** First 3 months of pregnancy	0	1	2	3	4	5	6	7	9	<30	30-60	>60	9
**ACT3.** Since the first 3 months of pregnancy	0	1	2	3	4	5	6	7	9	<30	30-60	>60	9

© Growing Up in New Zealand 2009

And how often would you have done Moderate exercise – which makes you breathe somewhat harder than normal and may include carrying light loads, bicycling at a regular pace, or doubles tennis?

Interviewer note: If respondent asks, clarify that exercise/activity at work is included.

ACT4A. Before you became pregnant, how many days per week (0 – 7) did you do moderate activity?

IF = 0 **GO TO ACT5A**

ACT4B. How long on average did you do this type of activity on these days (<30 mins, 30-60 min, more than 60 min)?

ACT5A. During the first 3 months of pregnancy, how many days per week (0 – 7) did you do moderate activity?

IF = 0 **GO TO ACT6A**

ACT5B. How long on average did you do this type of activity on these days (<30 mins, 30-60 min, more than 60 min)?

ACT6A. Since the first 3 months of pregnancy, how many days per week (0 – 7) did you do moderate activity?

IF = 0 **GO TO ACT7**

ACT6B. How long on average did you do this type of activity on these days (<30 mins, 30-60 min, more than 60 min)?

SHOWCARD ACT1B-6B

**Strenuous exercise**	**A. Average days per week circle ONE for each time period**									**B. Average length circle ONE for each**			
									DK/Ref	1	2	3	DK/Ref
**ACT4.** Pre-pregnancy	0	1	2	3	4	5	6	7	9	<30	30-60	>60	9
**ACT5.** First 3 months of pregnancy	0	1	2	3	4	5	6	7	9	<30	30-60	>60	9
**ACT6.** After the first 3 months of pregnancy	0	1	2	3	4	5	6	7	9	<30	30-60	>60	9

ACT7. Where do you do most of your exercise or physical activity? - Multi Response

SHOWCARD ACT7

In your home = 1
In your local area, but not in your home = 2
Outside of your local area = 3
At work = 4
Don't do any exercise = 5
DK/Ref = 9

© Growing Up in New Zealand 2009

Thinking about the last four weeks, approximately how many hours have you spent outdoors on an average……?

Interviewer Instruction: Must code last 4 weeks, even if abnormal.
Enter as double digits [RANGE: 0-18]

**ACT8.** Week day (Average number of hours)	
**ACT9.** Weekend day (Average number of hours)	

DO NOT READ OUT DK/Ref= 99

HEIGHT & WEIGHT

HW1. What is your height? Please estimate if you are unsure.

01 Specify cm **GO TO HW1A**
02 Specify feet and inches **GO TO HW1A**
DO NOT READ OUT 99 DK/Ref **GO TO HW2**

HW1A. Interviewer Instruction: Enter digits as required; 1m = 100cm [RANGE: cm 123-200; ft 4-6; in 0-11]. If don't know, an estimate is OK.

				Cm
Or		Ft		In

HW2. What was your weight before you became pregnant? Please estimate if you are unsure.

01 Specify Kilograms **GO TO HW2A**
02 Specify Stones/Pounds **GO TO HW2A**
03 Specify Pounds only **GO TO HW2A**
DO NOT READ OUT 99 DK/Ref **GO TO HW3**

HW2A. Interviewer Instruction: Enter as double or triple digits.
[RANGE: kg 38-200; st 6-32, lb 0-13; lb only; 84-440]

				Kg
Or		Stone		Lbs
Or				Lbs

HW3. What is your current weight at this stage in your pregnancy? Please estimate if you are unsure.

01 Specify Kilograms **GO TO HW3A**
02 Specify Stones/Pounds **GO TO HW3A**
03 Specify Pounds only **GO TO HW3A**
DO NOT READ OUT 99 DK/Ref **GO TO HW4**

HW3A. Interviewer Instruction: Enter as double or triple digits.
[RANGE: kg 38-200; st 6-32, lb 0-13; lb only; 84-440]

				Kg
Or		Stone		Lbs
Or				Lbs

SHOWCARD HW4

HW4. During this pregnancy have you:

Put on 5kg or more so far = 1
Put on weight, but less than 5 kg = 2
Stayed the same weight = 3
Lost weight, less than 5 kg = 4
Lost weight, 5 kg or more = 5
DK/Ref = 9

HW5. Over the 26 weeks – that is 6 months – prior to you becoming pregnant, were you actively dieting or trying to lose weight?

Yes = 1
No = 2 **GO TO NUT2**
DK/Ref = 9 **GO TO NUT2**

HW6. Did you lose any weight?

Yes = 1
No = 2 **GO TO NUT2**
DK/Ref = 9 **GO TO NUT2**

HW7. Approximately how much?

01 Specify Kilograms **GO TO HW7A**
02 Specify Pounds only **GO TO HW7A**
DO NOT READ OUT 99 DK/Ref **GO TO NUT2**

HW7A. Interviewer Instruction: Enter as double digits or triple as applicable
NB 14 Lbs = 1 Stone [RANGE: kg 1-24, lb 1-50] If less than 1 kg enter as 01 kg

		Kg
or		Lbs

NUTRITION

NUT2. Are there any **foods or drinks** you have deliberately avoided because of your pregnancy?

Yes =1
No = 2 **GO TO NUT4**
DK/Ref = 9 **GO TO NUT4**

SHOWCARD NUT3,5

NUT3. Could you please tell me the main foods or drinks you have deliberately **avoided**?
Interviewer Instruction: Probe to No. Record up to 5 items.

For each mention ask… Using this showcard could you please tell me which trimester of pregnancy you **avoided** consuming …(insert item).

	B.			
A. Food or drinks you **avoided consuming** during your pregnancy	During first 3 months of pregnancy (up to 12 weeks)	After first 3 months of pregnancy	Throughout the pregnancy	DK/Ref
1. Other 1	1	2	3	9
2. Other 2	1	2	3	9
3. Other 3	1	2	3	9
4. Other 4	1	2	3	9
5. Other 5	1	2	3	9

NUT4. Are there any foods or drinks you have **added** to your diet because of your pregnancy?

Yes =1
No = 2 **GO TO NUT6**
DK/Ref = 9 **GO TO NUT6**

SHOWCARD NUT3,5

NUT5. Could you please tell me the main foods or drinks you have **added**?
Interviewer Instruction: Probe to No. Record up to 5 items.

For each mention ask… Using this showcard could you please tell me which trimester of pregnancy you **added**…(insert item)

	B.			
A. Food or drinks you **added** during your pregnancy	During first 3 months of pregnancy (up to 12 weeks)	After first 3 months of pregnancy	Throughout the pregnancy	DK/Ref
1. Other 1	1	2	3	9
2. Other 2	1	2	3	9
3. Other 3	1	2	3	9
4. Other 4	1	2	3	9
5. Other 5	1	2	3	9

NUT6. <u>During this pregnancy</u> have you received any information or been told anything that has led you to make changes to what you are eating or drinking while you are pregnant? Include information received on becoming pregnant.

Yes = 1
No = 2 **GO TO NUT8**
DK/Ref = 9 **GO TO NUT8**

<u>SHOWCARD NUT7,8</u>

NUT7. Where did you get this information from?

Choose as many as apply
Family/whanau = 1
Friends = 2
GP (Family doctor) = 3
Midwife = 4
Obstetrician = 5
Dietician/nutritionist = 6
Alternative health practitioner = 7
Antenatal class = 8
The internet = 9
Radio = 10
TV = 11
Books, magazines, newspaper = 12
Other Please specify = 13
DK/Ref = 99

<u>SHOWCARD NUT7,8</u>

NUT8. Regardless of whether you have received any information or not – who or where would you trust to provide you with information about your diet during pregnancy

Choose as many as apply
Family/whanau = 1
Friends = 2
GP (Family doctor) = 3
Midwife = 4
Obstetrician = 5
Dietician/nutritionist = 6
Alternative health practitioner = 7
Antenatal class = 8
The internet = 9
Radio = 10
TV = 11
Books, magazines, newspaper = 12
Other Please specify = 13
DK/Ref = 99

I am going to ask you some questions about your usual eating patterns. When answering these questions please think back over the past four weeks…

SHOWCARD NUT9

NUT9. Firstly Fruit. Looking at this showcard please tell me which of the following you have eaten over the last 4 weeks.

Interviewer Instruction: Code all mentions

For each mention ask…
How many servings of …(insert variety) have you eaten over the past 4 weeks ?

Interviewer note: A serving is the same as a medium piece of fruit like an apple or two small pieces of fruit like two apricots, or half a cup of stewed fruit
Do not include fruit juice.
Showcard has examples of a serving of fruit.

**Fruit**	B. How often								
	per day				or per week			or per month	
	4+	3	2	1	5-6	3-4	1-2	2-3	1
01 Citrus fruits, such as oranges, lemons, grapefruit. Fresh, frozen, canned or stewed	☐	☐	☐	☐	☐	☐	☐	☐	☐
02 Non-citrus fruits, such as apricots, peaches, plums, apples, pears, bananas, mangoes, pawpaw, pineapple, melon, berries, kiwifruit, grapes. Fresh, frozen, canned or stewed	☐	☐	☐	☐	☐	☐	☐	☐	☐
03 Dried fruit	☐	☐	☐	☐	☐	☐	☐	☐	☐

Have eaten none of these = 04
DK/Ref = 99

© Growing Up in New Zealand 2009

SHOWCARD NUT10

NUT10. Now vegetables. Looking at this showcard please tell me which of the following you have eaten over the last 4 weeks.

Interviewer Instruction: Code all mentions

For each mention ask…
How many servings of …(insert variety) have you eaten over the past 4 weeks ?

Interviewer note: A serving is the same as one potato, half a cup of peas or a cup of salad.
Do not include vegetable juices

**Vegetables**	B. How often								
	per day				or per week			or per month	
	4+	3	2	1	5-6	3-4	1-2	2-3	1
01. Green leafy vegetables, such as lettuce, cabbage, bok choy, spinach. Fresh frozen or canned	☐	☐	☐	☐	☐	☐	☐	☐	☐
02. Other vegetables, such as avocado, broccoli, peas, carrots, tomatoes, green beans, mushrooms, peppers, potatoes, kumara, pumpkin, yams, taro. Fresh, frozen or canned	☐	☐	☐	☐	☐	☐	☐	☐	☐
03. Hot chips, french fries, wedges, or kumara chips	☐	☐	☐	☐	☐	☐	☐	☐	☐

Have eaten none of these = 04
DK/Ref = 99

SHOWCARD NUT11

NUT11. Now milk, cheese and yogurt. Looking at this showcard please tell me which of the following you have eaten or drunk over the last 4 weeks. When estimating milk intake you should include milk on cereal, milk added to beverages and milk as a drink (refer to showcard which shows a glass of milk as 1 serve and estimate number of these serves).

Interviewer Instruction: Code all mentions
For each mention ask…

How many servings of …(insert variety) have you eaten or drunk over the past 4 weeks

**Milk cheese and yoghurt**	B. How often								
	per day				or per week			or per month	
	4+	3	2	1	5-6	3-4	1-2	2-3	1
01. Whole or standard milk (Dark blue or silver)	☐	☐	☐	☐	☐	☐	☐	☐	☐
02. Reduced fat (light blue)	☐	☐	☐	☐	☐	☐	☐	☐	☐
03. Skim or Trim (Green or yellow	☐	☐	☐	☐	☐	☐	☐	☐	☐
04. Soy milk	☐	☐	☐	☐	☐	☐	☐	☐	☐
05. Other Milk (such as rice, goats milk)	☐	☐	☐	☐	☐	☐	☐	☐	☐
06. Cheese (including paneer, cottage cheese)	☐	☐	☐	☐	☐	☐	☐	☐	☐
07. Yoghurt	☐	☐	☐	☐	☐	☐	☐	☐	☐
08. Ice cream	☐	☐	☐	☐	☐	☐	☐	☐	☐

Have eaten/drunk none of these = 09
DK/Ref = 99

SHOWCARD NUT12

NUT12. Now bread, rice, pasta and cereals. Looking at this showcard please tell me which of the following you have eaten over the last 4 weeks.

Interviewer Instruction: Code all mentions
Interviewer note: If respondent is unsure whether their cereal is high fibre (code 06) or low fibre (code 07), code as low fibre (code 07).

For each mention ask…
How many servings of…(insert variety) have you eaten over the past 4 weeks

**Bread, rice, pasta, cereal**	B. How often										
	per day						or per week			or per month	
	6+	5	4	3	2	1	5-6	3-4	1-2	2-3	1
**Bread (or toast) slices or rolls**											
01. White	☐	☐	☐	☐	☐	☐	☐	☐	☐	☐	☐
02. High fibre white	☐	☐	☐	☐	☐	☐	☐	☐	☐	☐	☐
03. Brown bread, wholemeal or wholegrain	☐	☐	☐	☐	☐	☐	☐	☐	☐	☐	☐
04. Other bread, please specify	☐	☐	☐	☐	☐	☐	☐	☐	☐	☐	☐
**Rice, pasta**											
05. Noodles or rice or pasta	☐	☐	☐	☐	☐	☐	☐	☐	☐	☐	☐
**Breakfast cereal**											
06. High fibre cereals, such as muesli, porridge, weetbix, branflakes etc	☐	☐	☐	☐	☐	☐	☐	☐	☐	☐	☐
07. Other cereals, such as cornflakes, ricies, puffed wheat, nutrigrain etc	☐	☐	☐	☐	☐	☐	☐	☐	☐	☐	☐
08 Cakes or biscuits	☐	☐	☐	☐	☐	☐	☐	☐	☐	☐	☐

Have eaten/drunk none of these = 09
DK/Ref = 99

SHOWCARD NUT13

NUT13. Now spreads. Looking at this showcard please tell me which of the following you have eaten over the last 4 weeks. Do not include butter used in cooking or baking in this section.

Interviewer Instruction: Code all mentions

For each mention ask…
How many servings of…(insert variety) have you eaten over the past 4 weeks

**Spreads**	B. How often								
	per day				or per week			or per month	
	4+	3	2	1	5-6	3-4	1-2	2-3	1
01.Butter (including semi soft)	☐	☐	☐	☐	☐	☐	☐	☐	☐
02. Butter and margarine blend	☐	☐	☐	☐	☐	☐	☐	☐	☐
03. Margarine (Canola, sunflower, and olive oil and rice bran oil based)	☐	☐	☐	☐	☐	☐	☐	☐	☐
04. Lite or reduced fat margarine (Canola, sunflower, and olive oil and rice bran oil based)	☐	☐	☐	☐	☐	☐	☐	☐	☐
05. Plant sterol margarine (such as Proactive or Logical)	☐	☐	☐	☐	☐	☐	☐	☐	☐
06. Jam, honey, marmalade	☐	☐	☐	☐	☐	☐	☐	☐	☐
07. Peanut butter, Nutella	☐	☐	☐	☐	☐	☐	☐	☐	☐
08. Vegemite, Marmite	☐	☐	☐	☐	☐	☐	☐	☐	☐

Have eaten/drunk none of these = 09
DK/Ref = 99

SHOWCARD NUT14

NUT14. Now thinking about meat, alternative protein and eggs. Looking at this showcard please tell me which of the following you have eaten over the last 4 weeks

Interviewer Instruction: Code all mentions

For each mention ask…
How many servings of …(insert variety) have you eaten over the past 4 weeks

**Meat, meat alternatives, eggs**	B. How often								
	per day				or per week			or per month	
	4+	3	2	1	5-6	3-4	1-2	2-3	1
01. Red meat – such as beef, pork, mutton, lamb and goat	☐	☐	☐	☐	☐	☐	☐	☐	☐
02. Chicken – such as chicken breast, tenderloins, drumsticks, or whole chickens, but not chicken nuggets or chicken roll	☐	☐	☐	☐	☐	☐	☐	☐	☐
03. Processed meats – such as ham, bacon, pastrami, salami, sausages, luncheon, canned corned beef	☐	☐	☐	☐	☐	☐	☐	☐	☐
04. Seafood – such as fish or shellfish, fresh or frozen. Interviewer note: Do not include battered/fried or canned fish or seafood	☐	☐	☐	☐	☐	☐	☐	☐	☐
05. Battered or fried fish or seafood. Interviewer note: This may include battered or deep fried fish bought from the ‘Fish and Chip’ shop	☐	☐	☐	☐	☐	☐	☐	☐	☐
06. Processed such as tinned fish or fish sachets	☐	☐	☐	☐	☐	☐	☐	☐	☐
07. Takeaways from places like McDonalds, KFC, Burger King, Pizza shops or fast food outlets	☐	☐	☐	☐	☐	☐	☐	☐	☐
08. Alternative protein such as legumes, nuts, tofu, textured vegetable protein, vegetarian sausages or patties	☐	☐	☐	☐	☐	☐	☐	☐	☐
09. Eggs	☐	☐	☐	☐	☐	☐	☐	☐	☐

Have eaten none of these =10
DK/Ref = 99

© Growing Up in New Zealand 2009

SHOWCARD NUT15

NUT15. Soft drinks and snacks. Looking at this showcard please tell me which of the following you have eaten over the last 4 weeks.

Interviewer Instruction: Code all mentions

For each mention ask…
How many servings of …(insert variety) have you eaten over the past 4 weeks

Interviewer note: If necessary prompt "think about breakfast, lunch, dinner and snacks" for 01 and 02

**Soft drinks and snacks**	B. How often								
	per day				or per week			or per month	
	4+	3	2	1	5-6	3-4	1-2	2-3	1
01. Soft drinks or energy drinks. Soft drinks are often carbonated/ 'fizzy' and includes coca-cola, pepsi, lemonade, ginger beer, energy drinks ('V', Red Bull, Lift plus), Powerade, E2, G-force. This excludes – 'diet varieties', fruit juices and drinks, flavoured waters, and sports waters	☐	☐	☐	☐	☐	☐	☐	☐	☐
02. Fruit juices and drinks include freshly squeezed varieties, and brands such as Just Juice, Fresh-up, Keri, Golden Circle, Ribena, Thextons and Charlie's (includes spirulina). This Excludes – 'diet varieties', soft drinks, energy drinks, flavoured waters, and sports waters	☐	☐	☐	☐	☐	☐	☐	☐	☐
03. Soft drinks that don't contain sugar – this includes diet varieties	☐	☐	☐	☐	☐	☐	☐	☐	☐
04. Confectionary, lollies, sweets and chocolate?	☐	☐	☐	☐	☐	☐	☐	☐	☐
05. Snacks – crisps, nuts	☐	☐	☐	☐	☐	☐	☐	☐	☐

Have eaten none of these = 06
DK/Ref = 99

SHOWCARDS NUT16,17

Do you add salt to your food?

Do you add…	Never	Rarely (Less than once a week)	Occasionally (a few times a week)	Often (every or almost every day of the week)	DK/Ref **DO NOT READ OUT**
**NUT16.** Iodised table salt to your food?	0	1	2	3	9
**NUT17.** Rock salt or non-iodised table salt to your food?	0	1	2	3	9

Thinking about your health generally

SHOWCARD GH1
GH1. Thinking about before you became pregnant, in general would you say your health was

Poor = 0
Fair = 1
Good = 2
Very good = 3
Excellent = 4
DK/Ref = 9

GH2. Do you currently have a disability that is long term, lasting 6 months or more?
Interviewer note: code only current disabilities as "Yes"; if they have recovered from their disability, code as "No".

Yes = 1
No = 2 **GO TO GH4**
DK/Ref = 9 **GO TO GH4**

SHOWCARD GH3
GH3. How does this disability affect you? Multiple responses allowed

Hearing = 1
Seeing = 2
Speech = 3
Mobility = 4
Agility = 5
Intellectual function = 6
Psychiatric/psychological function = 7
Other Please specify = 8
DK/Ref = 99

Have you ever at any time in your life had any of the following illnesses diagnosed by a doctor?

SHOWCARD GH4-9

Have you ever had..(Insert illness)

If No Code 0 and ask for next illness
If DK/Ref Code 9 and ask for next illness
if Yes Code 1 and say….using **SHOWCARD GH4-9** when was this…

	NEVER	Before this pregnancy but not during this pregnancy	Before this pregnancy and during this pregnancy	Only during this current pregnancy	DK/Ref **DO NOT READ OUT**
**GH4.** Asthma	0	1	2	3	9
**GH5.** Anaemia	0	1	2	3	9
**GH6.** Depression	0	1	2	3	9
**GH7.** Either Heart disease or High blood pressure	0	1	2	3	9
**GH8.** Diabetes	0	1	2	3	9
**GH9.** Either Anxiety or Panic attacks	0	1	2	3	9

Vitamins, Minerals & Medications

Have you taken any vitamins or minerals **during your pregnancy or before you became pregnant**, e.g. folate or iron or multivitamins?

SHOWCARD VM1-3

First, thinking about folate or folic acid. Have you taken folate or folic acid, even as part of a multivitamin…

VM1. In the 3 months before you were pregnant ?
IF Yes How many days per week on average and for how many weeks:

Yes = 1
No = 2………If No….**GO TO VM2**
DK/Ref = 9………If DK….**GO TO VM2**

VM2. During the first 3 months of pregnancy?
IF Yes How many days per week on average and for how many weeks:

Yes = 1
No = 2………If No….**GO TO VM3**
DK/Ref = 9………If DK….**GO TO VM3**

VM3. Since the first 3 months of pregnancy?
IF Yes How many days per week on average and for how many weeks:

Yes = 1
No = 2………If No….**GO TO VM4**
DK/Ref = 9………If DK….**GO TO VM4**

SHOWCARD VM4-6

Next, thinking about iron. Have you taken iron, even as part of a multivitamin…

VM4. In the 3 months before you were pregnant?
IF Yes How many days per week on average and for how many weeks:

Yes = 1
No = 2………If No….**GO TO VM5**
DK/Ref = 9………If DK….**GO TO VM5**

VM5. During the first 3 months of pregnancy?
IF Yes How many days per week on average and for how many weeks:

Yes = 1
No = 2………If No….**GO TO VM6**
DK/Ref = 9………If DK….**GO TO VM6**

VM6. Since the first 3 months of pregnancy?
IF Yes How many days per week on average and for how many weeks:

Yes = 1
No = 2………If No….**GO TO VM7**
DK/Ref = 9………If DK….**GO TO VM7**

© Growing Up in New Zealand 2009

SHOWCARDS VM7-9

Now, thinking about vitamins or multivitamins and minerals. Have you taken vitamins or multivitamins and minerals, including those you may have already told me about…

VM7. In the 3 months before you were pregnant?
IF Yes How many days per week on average and for how many weeks:

Yes = 1
No = 2………If No….**GO TO VM8**
DK/Ref = 9………If DK….**GO TO VM8**

VM8. During the first 3 months of pregnancy?
IF Yes How many days per week on average and for how many weeks:

Yes = 1
No = 2………If No….**GO TO VM9**
DK/Ref = 9………If DK….**GO TO VM9**

VM9. Since the first 3 months of pregnancy?
IF Yes How many days per week on average and for how many weeks:

Yes = 1
No = 0………If No….**GO TO VM10**
DK/Ref = 9………If DK….**GO TO VM10**

IF ALL NOT TAKEN (=0) OR D/K (=9) TO VM1-VM9, GO TO VM11

Capsules, tablets, liquids or other preparations taken	A. Days per week taken					B. Number of weeks taken
	Not taken (0)	1 to 2 (1)	3 to 5 (2)	6 or 7 (3)	DK/Ref (9)	
**Folate or folic acid**						
**VM1.** In the 3 months before you were pregnant	☐	☐	☐	☐	☐	**[1-12, 13=13+]**
**VM2.** During the first 3 months of pregnancy	☐	☐	☐	☐	☐	**[1-12, 13=13+]**
**VM3.** Since the first 3 months of pregnancy	☐	☐	☐	☐	☐	**[1-25, 26=26+]**
**Iron**						
**VM4.** In the 3 months before you were pregnant	☐	☐	☐	☐	☐	**[1-12, 13=13+]**
**VM5.** During the first 3 months of pregnancy	☐	☐	☐	☐	☐	**[1-12, 13=13+]**
**VM6.** Since the first 3 months of pregnancy	☐	☐	☐	☐	☐	**[1-25, 26=26+]**
**Vitamins or multivitamins and minerals**						
**VM7.** In the 3 months before you were pregnant	☐	☐	☐	☐	☐	**[1-12, 13=13+]**
**VM8.** During the first 3 months of pregnancy	☐	☐	☐	☐	☐	**[1-12, 13=13+]**
**VM9.** Since the first 3 months of pregnancy	☐	☐	☐	☐	☐	**[1-25, 26=26+]**

DK/Ref = 99

SHOWCARDS VM10

VM10. Where did you get information about vitamins and minerals from?

Choose as many as apply
Family/whanau = 1
Friends = 2
GP (Family doctor) = 3
Midwife = 4
Obstetrician = 5
Dietician/nutritionist = 6
Alternative health practitioner = 7
Antenatal class = 8
The internet = 9
Radio = 10
TV = 11
Books, magazines, newspaper = 12
Other Please specify = 13
DK/Ref = 99

Now thinking about any medicines including over-the-counter medicine you may have taken during your pregnancy.

SHOWCARD VM11

Looking at this showcard, have you taken any medicines/inhalers/pills since you became pregnant?

CAPI: code these in variables VM11_1 – VM_16

Interviewer Instruction: None used, code 00; DK/Ref, code 99; Code all medicine types mentioned and for each mentioned say…

And using **SHOWCARD VM11A,C**,

A. For how many days per week (on average) did you take this medicine?

B. And for how many weeks was this medicine taken? **[RANGE 1-13 FOR VM11A_1, VM11A_2, VM11A_3 ... VM11A_16]**
[RANGE 1-26 FOR VM11C_1, VM11C_2, VM11C_3 ... VM11C_16]

Medication taken	Timeframe	A/C. Days per week taken					B/D. Number of weeks taken
		Not taken (0)	1 to 2 (1)	3 to 5 (2)	6 or 7 (3)	DK/ Ref (9)	
Antibiotics (01)	**VM11A_1.** 1st 3m of pregnancy	☐	☐	☐	☐	☐	
	**VM11C_1.** Since 1st 3m of pregnancy	☐	☐	☐	☐	☐	
Aspirin (02)	**VM11A_2.** 1st 3m of pregnancy	☐	☐	☐	☐	☐	
	**VM11C_2** Since 1st 3m of pregnancy	☐	☐	☐	☐	☐	
Paracetamol or Panadol (03)	**VM11A_3.** 1st 3m of pregnancy	☐	☐	☐	☐	☐	
	**VM11C_3.** Since 1st 3m of pregnancy	☐	☐	☐	☐	☐	
Anti-inflammatory medicines (04)	**VM11A_4.** 1st 3m of pregnancy	☐	☐	☐	☐	☐	
	**VM11C_4.** Since 1st 3m of pregnancy	☐	☐	☐	☐	☐	

Indigestion, heartburn or anti-reflux medicines (05)	**VM11A_5.** 1st 3m of pregnancy	☐	☐	☐	☐	☐	
	**VM11C_5.** Since 1st 3m of pregnancy	☐	☐	☐	☐	☐	

Asthma inhalers (06)	**VM11A_6.** 1st 3m of pregnancy	☐	☐	☐	☐	☐	
	**VM11C_6.** Since 1st 3m of pregnancy	☐	☐	☐	☐	☐	
Allergy medicines (07)	**VM11A_7.** 1st 3m of pregnancy	☐	☐	☐	☐	☐	
	**VM11C_7.** Since 1st 3m of pregnancy	☐	☐	☐	☐	☐	
Cough medicines (08)	**VM11A_8.** 1st 3m of pregnancy	☐	☐	☐	☐	☐	
	**VM11C_8.** Since 1st 3m of pregnancy	☐	☐	☐	☐	☐	
Diabetes medicines (09)	**VM11A_9.** 1st 3m of pregnancy	☐	☐	☐	☐	☐	
	**VM11C_9.** Since 1st 3m of pregnancy	☐	☐	☐	☐	☐	
Constipation medicines (10)	**VM11A_10.** 1st 3m of pregnancy	☐	☐	☐	☐	☐	
	**VM11C_10.** Since 1st 3m of pregnancy	☐	☐	☐	☐	☐	
Anti-depressants (11)	**VM11A_11.** 1st 3m of pregnancy	☐	☐	☐	☐	☐	
	**VM11C_11.** Since 1st 3m of pregnancy	☐	☐	☐	☐	☐	
Other medicine 1 Please Specify: (12)	**VM11A_12.** 1st 3m of pregnancy	☐	☐	☐	☐	☐	
	**VM11C_12.** Since 1st 3m of pregnancy	☐	☐	☐	☐	☐	
Other medicine 2 Please Specify: (13)	**VM11A_13.** 1st 3m of pregnancy	☐	☐	☐	☐	☐	
	**VM11C_13.** Since 1st 3m of pregnancy	☐	☐	☐	☐	☐	
Other medicine 3 Please Specify: (14)	**VM11A_14.** 1st 3m of pregnancy	☐	☐	☐	☐	☐	
	**VM11C_14.** Since 1st 3m of pregnancy	☐	☐	☐	☐	☐	
Other medicine 4 Please Specify: (15)	**VM11A_15.** 1st 3m of pregnancy	☐	☐	☐	☐	☐	
	**VM11C_15.** Since 1st 3m of pregnancy	☐	☐	☐	☐	☐	
Other medicine 5 Please Specify: (16)	**VM11A_16.** 1st 3m of pregnancy	☐	☐	☐	☐	☐	
	**VM11C_16.** Since 1st 3m of pregnancy	☐	☐	☐	☐	☐	

None used, code 00
DK/Ref, code 99

Now, thinking just about alcohol

And thinking about before you were pregnant, and during your pregnancy.

SHOWCARDS ALC1-3

ALC1. On average how many drinks of alcohol - beer, wine, spirits…

	**ALC1.** Did you drink per week before becoming pregnant or before you were aware you were pregnant	**ALC2.** Did you drink per week in the first 3 months of pregnancy	**ALC3.** Did you drink per week after the first 3 months of pregnancy
I did not drink alcohol	0	0	0
Less than 1 drink per week	1	1	1
1 drink per week	2	2	2
2 drinks per week	3	3	3
3 drinks per week	4	4	4
4-6 drinks	5	5	5
7 –9 drinks	6	6	6
10-14 drinks	7	7	7
15 – 19 drinks	8	8	8
20 to 39 drinks	9	9	9
40 or more drinks	10	10	10
**DO NOT READ OUT** DK/Ref	99	99	99

© Growing Up in New Zealand 2009

Now, questions about smoking

SM1. Did you smoke regularly - that is every day - **before you were aware you were pregnant**?

Yes = 1
No = 2 **GO TO SM4**
DK/Ref = 9 **GO TO SM4**

SM2. How many cigarettes did you smoke per day, on average, before this pregnancy?
Interviewer Instruction: Enter as double digits [RANGE: 1-80]

Cigarettes per day before pregnancy

Less than 1 per day = 0
DK/Ref = 99

SM3. At what age did you begin to smoke regularly, that is smoking every day?
Interviewer Instruction: Enter as double digits [RANGE: 5-50]
Spec Instruction: Use age at BG2 as upper age limit

Age in years

DK/Ref = 99

SM4. Are you **currently** smoking?

Yes = 1
No = 2 **GO TO SM6**
DK/Ref = 9 **GO TO SM6**

SM5. How many cigarettes do you smoke per day, on average?
Interviewer Instruction: Enter as double digits [RANGE: 1-80]

Cigarettes per day before pregnancy

Less than 1 per day = 0
DK/Ref = 99

SM6. Does anyone currently regularly smoke in the same room as you?

Yes = 1
No = 2 **GO TO SECTION B**
DK/Ref = 9 **GO TO SECTION B**

SHOWCARD SM7

SM7. How often?

Rarely (Less than once a week) = 1
Occasionally (a few times a week) = 2
Often (almost or every day of the week) = 3
DK/Ref = 9

Section B: Psychological & Cognitive

People's thoughts and feelings often differ when they are pregnant.
I would like to ask you some questions about how things have been going for you in the last seven days. Although some of the questions seem similar, there are differences between them, so please treat each one as a separate question. Please read out the number which comes closest to how you have felt in the past 7 days – not just how you feel today.

SHOWCARD EDI1

EDI1. So, in the last 7 days I have been able to laugh and see the funny side of things:

As much as I always could = 0
Not quite so much now = 1
Definitely not so much now = 2
Not at all = 3
DO NOT READ OUT DK/Ref = 9

SHOWCARD EDI2

EDI2. In the last 7 days I have blamed myself for no particular reason when things went wrong:

Yes, most of the time = 3
Yes, some of the time = 2
Not very often = 1
No, never = 0
DO NOT READ OUT DK/Ref = 9

SHOWCARD EDI3

EDI3. In the last 7 days I have been anxious or worried for no particular reason:

No, not at all = 0
Hardly ever = 1
Yes, sometimes = 2
Yes, very often = 3
DO NOT READ OUT DK/Ref = 9

SHOWCARD EDI4

EDI4. In the last 7 days I have felt scared or panicky for no particular reason:

Yes, quite a lot = 3
Yes, sometimes = 2
No, not much = 1
No, not at all = 0
DO NOT READ OUT DK/Ref = 9

© Growing Up in New Zealand 2009

SHOWCARD EDI5

EDI5. In the last 7 days things have been getting too much for me:

Yes, most of the time I haven't been able to cope at all = 3
Yes, sometimes I haven't been coping as well as usual = 2
No, most of the time I have coped quite well = 1
No, I have been coping as well as ever = 0
DO NOT READ OUT DK/Ref = 9

SHOWCARD EDI6

EDI6. In the last 7 days I have been so unhappy that I have had difficulty sleeping:

Yes, most of the time = 3
Yes, sometimes = 2
Not very often = 1
No, not at all = 0
DO NOT READ OUT DK/Ref = 9

SHOWCARD EDI7

EDI7. In the last 7 days I have felt sad or miserable:

Yes, most of the time = 3
Yes, quite often = 2
Not very often = 1
No, not at all = 0
DO NOT READ OUT DK/Ref = 9

SHOWCARD EDI8

EDI8. In the last 7 days the thought of harming myself has occurred to me:

Yes, quite often = 3
Sometimes = 2
Hardly ever = 1
Never = 0
DO NOT READ OUT DK/Ref = 9

SHOWCARD EDI9

EDI9. In the last 7 days I have been so unhappy that I have been crying:

Yes, most of the time = 3
Yes, quite often = 2
Only occasionally = 1
No, never = 0
DO NOT READ OUT DK/Ref = 9

© Growing Up in New Zealand 2009

SHOWCARD EDI10

EDI10. In the last 7 days I have looked forward with enjoyment to things:

As much as I ever did = 0
Rather less than I used to = 1
Definitely less than I used to = 2
Hardly at all = 3
DO NOT READ OUT DK/Ref = 9

Optional interviewer comment if the person found the questionnaire a bit odd or upsetting:
You can explain that this questionnaire is used internationally in similar studies and to make comparisons we need to ask the same questions.

The last set of questions asked about your thoughts and feelings over the last week. I would now like to ask you about your feelings and thoughts over the last four weeks. In each case you will be asked *how often* you felt or thought in a certain way. Although some of the questions seem similar, there are differences between them, so please treat each one as a separate question.

SHOWCARD PSS1-10

PSS1. In the last four weeks, how often have you been upset because of something that happened unexpectedly?

Never	Almost never	Sometimes	Fairly often	Very often	DK/Ref **DO NOT READ OUT**
0	1	2	3	4	9

PSS2. In the last four weeks, how often have you felt that you were unable to control the important things in your life?

Never	Almost never	Sometimes	Fairly often	Very often	DK/Ref **DO NOT READ OUT**
0	1	2	3	4	9

PSS3. In the last four weeks, how often have you felt nervous and stressed?

Never	Almost never	Sometimes	Fairly often	Very often	DK/Ref **DO NOT READ OUT**
0	1	2	3	4	9

PSS4. In the last four weeks, how often have you felt confident about your ability to handle your personal problems?

Never	Almost never	Sometimes	Fairly often	Very often	DK/Ref **DO NOT READ OUT**
0	1	2	3	4	9

© Growing Up in New Zealand 2009

PSS5. In the last four weeks, how often have you felt that things were going your way?

Never	Almost never	Sometimes	Fairly often	Very often	DK/Ref **DO NOT READ OUT**
0	1	2	3	4	9

PSS6. In the last four weeks, how often have you found that you could not cope with all the things that you had to do?

Never	Almost never	Sometimes	Fairly often	Very often	DK/Ref **DO NOT READ OUT**
0	1	2	3	4	9

PSS7. In the last four weeks, how often have you been able to control the irritations in your life?

Never	Almost never	Sometimes	Fairly often	Very often	DK/Ref **DO NOT READ OUT**
0	1	2	3	4	9

PSS8. In the last four weeks, how often have you felt that you were on top of things?

Never	Almost never	Sometimes	Fairly often	Very often	DK/Ref **DO NOT READ OUT**
0	1	2	3	4	9

PSS9. In the last four weeks, how often have you been angered because of things that were outside of your control?

Never	Almost never	Sometimes	Fairly often	Very often	DK/Ref **DO NOT READ OUT**
0	1	2	3	4	9

PSS10. In the last four weeks, how often have you felt difficulties were so great that you could not overcome them?

Never	Almost never	Sometimes	Fairly often	Very often	DK/Ref **DO NOT READ OUT**
0	1	2	3	4	9

© Growing Up in New Zealand 2009

Section C: Questions about your Family or Whānau

This section is about your household and your family members…

HH1. How long have you lived in this current home?

Specify years and months = 01 **GO TO HH1A**
Specify months only = 02 **GO TO HH1A**
DO NOT READ OUT DK/Ref = 99 **GO TO HH2**

HH1A. Interviewer Instruction: Enter as double digits. If Respondent is unsure of exact time frame enter what is known e.g. 9 years as 09 years 00 months; If less than 1 month, enter as 01 months [RANGE: years<= CAPI-generated age from BG2; Month only range as 01-24 months]

- - years - - months
OR - - months

HH2. How many people are in the household, **not counting yourself**?

Code number
99 = DK/Ref
If code =0 Skip to HH3A

SHOWCARD HH2B

HH2A. Please list all the people who live in this place/dwelling and then indicate how they are related to you
Interviewer Instruction: Enter name on grid under Name, and enter relationship code in Rel column. If other, specify relationship

Code	HH2A: Name	HH2B: Rel
1		
2		
3		
4		
5		
6		
7		
8		
9		
10		
11		
12		
13		
14		
15		
	**SPEC INSTRUCTION: Check Code 1 Partner and Code 2 Wife/Husband. Together must appear maximum of TWICE at Relationship**	**1 = PARTNER<br>2 = WIFE/HUSBAND<br>3 = SON<br>4 = DAUGHTER<br>5 = MOTHER<br>6 = FATHER<br>7 = AUNTIE<br>8 = UNCLE<br>9 = COUSIN<br>10 = GRANDMOTHER<br>11 = GRANDFATHER<br>12 = FLATMATE<br>13 = BOARDER<br>14 = SISTER<br>15 = BROTHER<br>16 = SISTER IN LAW<br>17 = BROTHER IN LAW<br>18 = GRANDCHILD<br>19 = MOTHER IN LAW<br>20 = FATHER IN LAW<br>97 = OTHER, PLEASE SPECIFY**

© Growing Up in New Zealand 2009

I'm now going to ask some questions about people you consider to be members of your family or whanau. They may live with you and be on the list we have just made, or they may live somewhere else. They might not be related biologically to you.

Interviewer note: Use "Family" or "Whānau" as indicated by respondent. If respondent asks about deceased family members, explain that these should not be included.

HH3A. First, do you have a spouse or partner whom you consider to be a member of your family/whānau?

No = 0
Yes = 1
DK/Ref = 9

Next, how many…

HH3B. brothers and sisters do you have who you consider to be members of your family/ whānau? This may include step- and half-brothers and sisters, and brothers and sisters-in-law._________

HH3C. parents do you have who you consider to be members of your family/ whānau? This may include in-laws and foster parents. _________

HH3D. grandparents do you have who you consider to be members of your family/ whānau?_________

HH3E. aunts and uncles do you have who you consider to be members of your family/ whānau? _________

HH3F. nieces and nephews do you have who you consider to be members of your family/ whānau? _________

HH3G. cousins do you have who you consider to be members of your family/ whānau? _________

HH3H. grandchildren do you have who you consider to be members of your family/ whānau?_________

HH3I. children do you have who you consider to be members of your family/ whānau? This may include step or foster children. _________

HH3J. and are there any other people you have not already mentioned who you consider to be members of your family/ whānau, for example people that are not related to you? How many?

Interviewer instruction- Friends whom they consider to be members of their family SHOULD be included in this category _________

For HH3B-HH3J code 0 if none, number (1-30) or 99 if DK/Ref.

© Growing Up in New Zealand 2009

We are interested in how New Zealand families or whānau think about each other.

Thinking about the members of your family/ whānau we have just talked about, to what extent do the following statements apply?

<u>Interviewer Note</u>: Use wording "Family" or" Whānau" as indicated by respondent.

<u>SHOWCARD COH1-9</u>

COH1. People in our family **/** whānau ask each other for help, when they need it.

Never	Sometimes	Usually	Always	DK/Ref **DO NOT READ OUT**
1	2	3	4	9

COH2. When someone does something good for our family, we try to do something back for that person.

Never	Sometimes	Usually	Always	DK/Ref **DO NOT READ OUT**
1	2	3	4	9

COH3. There are times when our family enjoys doing activities that are just with our family / whānau.

Never	Sometimes	Usually	Always	DK/Ref **DO NOT READ OUT**
1	2	3	4	9

COH4. People in our family / whānau would provide for each other even if there is very little to go around.

Never	Sometimes	Usually	Always	DK/Ref **DO NOT READ OUT**
1	2	3	4	9

© Growing Up in New Zealand 2009

COH5. We feel very close to each other in our family / whānau.

Never	Sometimes	Usually	Always	DK/Ref **DO NOT READ OUT**
1	2	3	4	9

COH6. People in our family / whānau support each other at difficult times.

Never	Sometimes	Usually	Always	DK/Ref **DO NOT READ OUT**
1	2	3	4	9

COH7. When our family / whānau has an important activity such as a wedding or hui, everyone tries to be present.

Never	Sometimes	Usually	Always	DK/Ref **DO NOT READ OUT**
1	2	3	4	9

COH8. We can easily think of things to do together as a family / whānau group.

Never	Sometimes	Usually	Always	DK/Ref **DO NOT READ OUT**
1	2	3	4	9

COH9. We ask each other for advice about important decisions in our family / whānau.

Never	Sometimes	Usually	Always	DK/Ref **DO NOT READ OUT**
1	2	3	4	9

© Growing Up in New Zealand 2009

SHOWCARD SPF1-SPE6

I am going to list some sources that are sometimes helpful to caregivers raising young children. Please indicate how helpful you **EXPECT** each source of support to be when your baby is born.
If any of these sources are not available to you, choose 'Not Available'.

How helpful do you expect ….(insert source of help) to be generally?

		**Helpfulness** Please circle ONE per source					
	**Source of help**	Not available	Not at all helpful	Sometimes helpful	Generally helpful	Very helpful	Extremely helpful
**SPF1**	Your partner	1	2	3	4	5	6
**SPF2**	Your parent/s	1	2	3	4	5	6
**SPF3**	Your partner's parent/s	1	2	3	4	5	6
**SPF4**	Your extended family (cousins, brothers and sisters, grandparents, etc)	1	2	3	4	5	6
**SPF5**	Your partner's extended family (cousins, grandparents, brothers and sisters, etc)	1	2	3	4	5	6
**SPF6**	Your friends	1	2	3	4	5	6
**SPE1**	Your family doctor	1	2	3	4	5	6
**SPE2**	Professionals (e.g Plunket nurse, kaiawhina)	1	2	3	4	5	6
**SPE3**	Kindy, Preschool, day care, Kohanga Reo etc.	1	2	3	4	5	6
**SPE4**	Early parenting support programmes e.g. Parents as First Teacher	1	2	3	4	5	6
**SPE5**	Books	1	2	3	4	5	6
**SPE6**	Internet	1	2	3	4	5	6

If DK/Ref Code 9

To what extent are the following a source of stress for you and your family

SHOWCARD FS1-6

FS1. Worry about a disabled or ill family member:

Not at all stressful	Somewhat stressful	Moderately stressful	Highly stressful	Not Applicable	DK/Ref **DO NOT READ OUT**
1	2	3	4	5	9

FS2. Worry about current housing difficulties:

Not at all stressful	Somewhat stressful	Moderately stressful	Highly stressful	Not Applicable	DK/Ref **DO NOT READ OUT**
1	2	3	4	5	9

FS3. Worry about balancing work and family life:

Not at all stressful	Somewhat stressful	Moderately stressful	Highly stressful	Not Applicable	DK/Ref **DO NOT READ OUT**
1	2	3	4	5	9

FS4. Worry about money problems:

Not at all stressful	Somewhat stressful	Moderately stressful	Highly stressful	Not Applicable	DK/Ref **DO NOT READ OUT**
1	2	3	4	5	9

FS5. Worry about family members not getting on:

Not at all stressful	Somewhat stressful	Moderately stressful	Highly stressful	Not Applicable	DK/Ref **DO NOT READ OUT**
1	2	3	4	5	9

FS6. Worry about another child's behaviour:

Not at all stressful	Somewhat stressful	Moderately stressful	Highly stressful	Not Applicable	DK/Ref **DO NOT READ OUT**
1	2	3	4	5	9

© Growing Up in New Zealand 2009

This section includes Questions about your relationship

REL1. Do you have a current partner?

Yes = 1 (**GO TO REL2**)
No = 2 (**GO TO REL8**)
DK/Ref =9 (**GO TO REL8**)

Interviewer note: refer to "partner" or "husband" as indicated by the respondent.
If Response at REL 1 is No = 2, a check screen will appear to ensure this response is data entered correctly. Simply enter the response again or correct if necessary, without re-asking the question.

SHOWCARD REL2

REL2. What best describes the nature of your relationship with your current partner?

Dating AND not cohabiting (Not living together) = 1 **GO TO REL7**
Cohabiting (de facto) (Living together) = 2 **GO TO REL6**
Married = 3 **GO TO REL3**
Civil Union = 4 **GO TO REL3**
DO NOT READ OUT DK/Ref = 9 **GO TO REL8**

REL3. How long have you been married / in a civil union?

Specify years and months = 01 **GO TO REL3A**
Specify months only = 02 **GO TO REL3A**
DO NOT READ OUT DK/Ref = 99 **GO TO REL4**

REL3A. **Interviewer Instruction: Enter as many digits as required. If less than 1 month Code as 01 Months**
[RANGE: years & months, years=01-30; months=00-11; months only=01-24]

	Number of years		Number of months
OR			Number of months

REL4. And did you live together before marrying / entering a civil union?

Yes = 1 (**GO TO REL5**)
No = 2 (**GO TO REL8**)
DK/Ref = 9 (**GO TO REL8**)

REL5. For how long?

Specify years and months = 01 **GO TO REL5A**
Specify months only = 02 **GO TO REL5A**
DO NOT READ OUT DK/Ref = 99 **GO TO REL8**

REL5A. **Interviewer Instruction: Enter as many digits as required. If less than 1 month Code as 01 Months**
[RANGE: years & months, years=01-30; months=00-11; months only=01-24]

	Number of years		Number of months
OR			Number of months

NOW GO TO REL8

REL6. How long have you lived together?

Specify years and months = 01 **GO TO REL6A**
Specify months only = 02 **GO TO REL6A**
DO NOT READ OUT DK/Ref = 99 **GO TO REL8**

REL6A. **<u>Interviewer Instruction</u>: Enter as many digits as required. If less than 1 month Code as 01 Months**
[RANGE: years & months, years=01-30; months=00-11; months only=01-24]

	Number of years		Number of months
OR			Number of months

NOW GO TO REL8

REL7. How long have you been in this relationship?

Specify years and months = 01 **GO TO REL7A**
Specify months only = 02 **GO TO REL7A**
DO NOT READ OUT DK/Ref = 99 **GO TO REL8**

REL7A. **<u>Interviewer Instruction</u>: Enter as many digits as required If less than 1 month Code as 01 Months**
[RANGE: years & months, years=01-30; months=00-11; months only=01-24]

	Number of years		Number of months
OR			Number of months

<u>SHOWCARD REL8</u>

REL8. What was your relationship with your baby's biological father <u>at the time you became pregnant</u>?

Married = 1 **GO TO REL10**
Cohabiting (Living together) = 2 **GO TO REL10**
A couple but not living together = 3 **GO TO REL10**
Dating (i.e. 'going out' but not living together) = 4 **GO TO REL10**
No relationship = 5 **GO TO REL9**
DK/Ref = 9 **GO TO REL10**

REL9. What were the circumstances of your becoming pregnant, e.g. casual encounter, donor insemination? MULTI RESPONSE

<u>SHOWCARD REL9</u>

Casual Encounter =01
Donor Insemination = 02
Other please specify___________97
DO NOT READ OUT DK/Ref = 99

REL10. Thinking about now, is your relationship with your baby's biological father the same as it was at the time you became pregnant?

Yes = 1 **GO TO WH1**
No = 2 **GO TO REL11**
DO NOT READ OUT DK/Ref = 9 **GO TO WH1**

SHOWCARD REL11

REL11. If no, what has changed?

Separated, not in another relationship = 1
Separated, in another relationship = 2
Cohabiting/living together (previously dating) = 3
Living apart but a couple (previously cohabiting/living together) = 4
Married (previously dating or cohabiting/living together) = 5
Engaged (previously dating or cohabiting/living together) = 6
Other Please specify (OPTION DOES NOT APPEAR ON SHOWCARD) = 7
DK/Ref = 9

The next section is about You and Your Partner

Please answer the questions in this section with regard to your current partner.

[If no current partner – from REL1 (= 2) or REL11 (= 1) GO TO INV1]

SHOWCARD WH1-CFL6

Please think about the time during the past four weeks when you and your partner have spent time talking or doing things together. With those times in mind, please select the number on the showcard that tells how often you acted in the following way towards each other during the past four weeks.

During the past four weeks how often did you…

		**How have often you acted this way towards each other** Please circle ONE number for each						
		All the time	Extremely often	Very often	Quite often	Not very often	Almost never	Never
**WH1.**	Let each other know you really care about each other	1	2	3	4	5	6	7
**WH2.**	Get angry with each other	1	2	3	4	5	6	7
**WH3.**	Dislike each other's ideas	1	2	3	4	5	6	7
**WH4.**	Shout at each other because you were upset with each other	1	2	3	4	5	6	7
**WH5.**	Act lovingly and affectionately towards each other	1	2	3	4	5	6	7
**WH6.**	Let each other know that you appreciate each other's ideas or the things you do	1	2	3	4	5	6	7
**WH7.**	Help the other do something that was important to her/him	1	2	3	4	5	6	7
**WH8.**	Argue with each other when you disagree about something	1	2	3	4	5	6	7
**WH9.**	Act supportive and understanding towards each other	1	2	3	4	5	6	7
**CFL1.**	Push and shove each other when arguing	1	2	3	4	5	6	7
**CFL2.**	Raise your voices when arguing	1	2	3	4	5	6	7
**CFL3.**	Throw things at each other when arguing	1	2	3	4	5	6	7
**CFL4.**	Yell at each other when angry	1	2	3	4	5	6	7
**CFL5.**	Break things when arguing	1	2	3	4	5	6	7
**CFL6.**	Swear at each other when angry	1	2	3	4	5	6	7

If DK/Ref Code 9

Please indicate the extent to which you agree with these statements:

SHOWCARD CMT1-6

CMT1. I want to grow old with my partner.

Strongly disagree	Mildly disagree	Not sure	Agree	Strongly agree	DK/Ref **DO NOT READ OUT**
1	2	3	4	5	9

CMT2. When I imagine what my life will be like in the future I always see my partner standing next to me.

Strongly disagree	Mildly disagree	Not sure	Agree	Strongly agree	DK/Ref **DO NOT READ OUT**
1	2	3	4	5	9

CMT3. Even when things get hard it is important to work through difficulties for the sake of the relationship.

Strongly disagree	Mildly disagree	Not sure	Agree	Strongly agree	DK/Ref **DO NOT READ OUT**
1	2	3	4	5	9

CMT4. When we agree to be together and have children, we should expect to stay together.

Strongly disagree	Mildly disagree	Not sure	Agree	Strongly agree	DK/Ref **DO NOT READ OUT**
1	2	3	4	5	9

CMT5. The shame or disapproval of separation would stop me separating from my partner.

Strongly disagree	Mildly disagree	Not sure	Agree	Strongly agree	DK/Ref **DO NOT READ OUT**
1	2	3	4	5	9

CMT6. The material costs of separation, for example housing costs, lower income, would stop me from separating from my partner.
Interviewer note: material costs mean financial costs

Strongly disagree	Mildly disagree	Not sure	Agree	Strongly agree	DK/Ref **DO NOT READ OUT**
1	2	3	4	5	9

We are interested in the plans you and your partner may have about being involved with your baby after they are born. Please tell me the number on the showcard for the response that best represents your expectations.

SHOWCARD INV1

INV1. To what extent do you hope to be involved in the day to day care of your baby, for example feeding, holding, changing?

All of the time	Most of the time	Some of the time	Not much of the time	DK/Ref **DO NOT READ OUT**
4	3	2	1	9

SHOWCARD INV2

Interviewer note: code 0 if no partner

INV2. How involved do you expect your ***partner*** will be in the day to day care of your baby?

All of the time	Most of the time	Some of the time	Not much of the time	Not Applicable	DK/Ref **DO NOT READ OUT**
4	3	2	1	0	9

SHOWCARD INV3

INV3. How much of the time do you expect to be **directly responsible** for your baby, for example, in sole care of him/her, making babysitting arrangements, looking after him/her if they are sick?

All of the time	Most of the time	Some of the time	Not much of the time	DK/Ref **DO NOT READ OUT**
4	3	2	1	9

SHOWCARD INV4

Interviewer note: code 0 if no partner

INV4. How often do you expect that your ***partner*** will **be directly responsible** for your baby?

All of the time	Most of the time	Some of the time	Not much of the time	Not Applicable	DK/Ref **DO NOT READ OUT**
4	3	2	1	0	9

SHOWCARD INV5

INV5. We would like to know how you are feeling about being the parent of this baby
Overall, do you feel that as a parent you will be: READ OUT Single Response

Not very good at being a parent = 1
A person who has some trouble being a parent = 2
An average parent = 3
A better than average parent = 4
A very good parent = 5
DO NOT READ OUT DK/Ref **=** 9

© Growing Up in New Zealand 2009

Section D: Culture & Identity

The next set of questions ask about who you are - your own cultural identity, where you were born, cultural activities you engage in – from 'kiwi' or "mainstream" culture to other 'ethnic' cultures. Please take a few moments to consider what it is in your life that makes you feel you belong to a particular cultural or ethnic group.

Please think about this when you answer the following questions. SINGLE RESPONSE

SHOWCARD ETH1

ETH1. Which country were you born in?

New Zealand = 1 **GO TO ETH3**
Australia = 2
Samoa = 3
Cook Islands = 4
Fiji = 5
Tonga = 6
United Kingdom (includes England, Scotland, Wales, Northern Ireland) = 7
Niue = 8
China (People's Republic of) = 9
South Africa = 10
Korea = 11
Hong Kong = 12
India = 13
Sri Lanka = 14
Malaysia = 15
Indonesia = 16
Japan = 17
Europe = 18
Middle East = 19
North America = 20
South America = 21
Africa = 22
Another country Please specify = 97
DK/Ref = 99

ETH2. Ask if NOT Code 1 at **ETH1.** When did you first arrive **to live** in New Zealand?

DK DO NOT READ OUT = 99
REFUSED DO NOT READ OUT = 98

ETH2A. ENTER DATE. IF ONLY AN APPROX DATE OR KNOW JUST YEAR ENTER WHAT IS KNOWN (e.g. 00 00 2004)
[RANGE: yyyy>= 1949; < interview date] SPEC CHECK: Check that date entered doesn't exceed current date and is greater than or equal to respondent's year of birth at BG1

d	d	m	m	y	y	y	y

SHOWCARD ETH3-4

ETH3. Which ethnic group OR GROUPS do you belong to? (Choose the answer or answers that apply to you)

Multi response

New Zealand European	1
Māori	2
Samoan	3
Cook Islands Maori	4
Tongan	5
Niuean	6
Tokelauan	7
Fijian	8
Fijian Indian	9
Other Pacific Peoples	10
Indian	11
Sri Lankan	12
Other Asian	13
Chinese	14
Korean	15
Japanese	16
Filipino	17
Cambodian	18
Vietnamese	19
Other Southeast Asian	20
Australian	21
British and Irish	22
Dutch	23
Greek	24
Polish	25
South Slav (formerly Yugoslav)	26
Italian	27
German	28
Other European	29
Middle Eastern	30
Latin American/Hispanic	31
African	32
New Zealander **NOT ON CARD**	40
Other Ethnicity Please specify	96
Other Ethnicity Please specify	97
DK/Ref	99

SHOWCARD ETH3-4

ETH4. Which is your **main** ethnic group that is the one you identify with most?

Interviewer note: Code maximum of 2 responses. Only use other box if not on code frame. If two 'other' mentions use separate other boxes. e.g. Native American Code 96, Inuit Code 97

New Zealand European	1
Māori	2
Samoan	3
Cook Islands Maori	4
Tongan	5
Niuean	6
Tokelauan	7
Fijian	8
Fijian Indian	9
Other Pacific Peoples	10
Indian	11
Sri Lankan	12
Other Asian	13
Chinese	14
Korean	15
Japanese	16
Filipino	17
Cambodian	18
Vietnamese	19
Other Southeast Asian	20
Australian	21
British and Irish	22
Dutch	23
Greek	24
Polish	25
South Slav (formerly Yugoslav)	26
Italian	27
German	28
Other European	29
Middle Eastern	30
Latin American/Hispanic	31
African	32
New Zealander **NOT ON CARD**	40
Other Ethnicity Please specify	96
Other Ethnicity Please specify	97
DK/Ref	99

SHOWCARD LAN1-4

LAN1. In which language(s) could you have a conversation about a lot of everyday things?
Multi response: Code all that apply

English = 1
Maori = 2
Samoan = 3
Tongan = 4
Fijian = 5
Niuean = 6
Cook Islands Maori = 7
Cantonese = 8
Mandarin = 9
Korean = 10
Japanese = 11
Hindi = 12
Arabic = 13
Other please specify = 97
DO NOT READ OUT DK/Ref **=** 99

SHOWCARD LAN1-4

LAN2. What language do you usually speak at home? If you speak more than one language, choose the one you speak most.
Single response

English = 1
Maori = 2
Samoan = 3
Tongan = 4
Fijian = 5
Niuean = 6
Cook Islands Maori = 7
Cantonese = 8
Mandarin = 9
Korean = 10
Japanese = 11
Hindi = 12
Arabic = 13
Other please specify = 97
DO NOT READ OUT DK/Ref **=** 99

SHOWCARD LAN1-4

LAN3. Which language were you expected to speak as a child when you were at home?
Single response

English = 1
Maori = 2
Samoan = 3
Tongan = 4
Fijian = 5
Niuean = 6
Cook Islands Maori = 7
Cantonese = 8
Mandarin = 9
Korean = 10
Japanese = 11
Hindi = 12
Arabic = 13
Other please specify = 97
DO NOT READ OUT DK/Ref **=** 99

SHOWCARD LAN1-4

LAN4. When you were at school, what was the FIRST language in which you learned to read and write?
Single response

English = 1
Maori = 2
Samoan = 3
Tongan = 4
Fijian = 5
Niuean = 6
Cook Islands Maori = 7
Cantonese = 8
Mandarin = 9
Korean = 10
Japanese = 11
Hindi = 12
Arabic = 13
Other please specify = 97
DO NOT READ OUT DK/Ref **=** 99

In recent years there has been a growing awareness of a distinct New Zealand identity – things which make us unique as Kiwis and which separate us from other countries. The All Blacks, Bar-B-Q's, Waitangi Day Commemorations, or even the Māori New-Year "Matariki" are just some examples of these. The following series of questions is designed to explore your knowledge of Kiwi culture. Remember, there are no right or wrong answers, and each response should be based on your idea of what Kiwi culture is.

SHOWCARD IDQ1
IDQ1. How **knowledgeable** are you of Kiwi/New Zealand culture and lifestyle?

Very knowledgeable	Fairly knowledgeable	Somewhat knowledgeable	Not very knowledgeable	Not at all knowledgeable	DK/Ref **DO NOT READ OUT**
1	2	3	4	5	9

SHOWCARD IDQ2
IDQ2. How **involved** are you in Kiwi/New Zealand culture and lifestyle?

Very involved	Fairly involved	Somewhat involved	Not involved much	Not involved at all	DK/Ref **DO NOT READ OUT**
1	2	3	4	5	9

SHOWCARD IDQ3
IDQ3. How do you **feel** toward Kiwi/New Zealand culture and lifestyle?

Very positive	Fairly positive	Neither positive nor negative	Slightly negative	Very negative	DK/Ref **DO NOT READ OUT**
1	2	3	4	5	9

SHOWCARD IDQ4
IDQ4. How often do you **associate** with Kiwis/New Zealanders?

Most of the time	Often	Sometimes	Not often	Almost never	DK/Ref **DO NOT READ OUT**
1	2	3	4	5	9

SHOWCARD IDQ5
IDQ5. How important is it to **maintain** a Kiwi/New Zealand culture and lifestyle?

Very important	Fairly important	Somewhat important	Not very important	Not important at all	DK/Ref **DO NOT READ OUT**
1	2	3	4	5	9

© Growing Up in New Zealand 2009

If only ethnic group is NZ European/New Zealander (If only Code 1 or 40 or 99 at ETH3-skip IDQ6-12 GO TO IDQ13)

If Response at ETH3 does not include Code 1 or 40 say….
The following series of questions relate to the ethnic group(s) you identified with previously. It is important that you complete these questions to the best of your knowledge and with your ethnic group(s) in mind.

OR Use this intro if **NZ European/New Zealander Code 1 or 40 at ETH3 AND another code mentioned at ETH3**

The following series of questions relate to the ethnic group you identified with previously along with **NZ European/New Zealander**. It is important that you complete these questions to the best of your knowledge and with your other ethnic group in mind not NZ European/New Zealander.

SHOWCARD IDQ6
IDQ6. How **knowledgeable** are you of your traditional culture?

Very knowledgeable	Fairly knowledgeable	Somewhat knowledgeable	Not very knowledgeable	Not at all knowledgeable	DK/Ref **DO NOT READ OUT**
1	2	3	4	5	9

SHOWCARD IDQ7
IDQ7. How **involved** are you in your traditional cultural activities?

Very involved	Fairly involved	Somewhat involved	Not involved much	Not involved at all	DK/Ref **DO NOT READ OUT**
1	2	3	4	5	9

SHOWCARD IDQ8
IDQ8. How do you **feel** about your culture?

Very positive	Fairly positive	Neither positive nor negative	Slightly negative	Very negative	DK/Ref **DO NOT READ OUT**
1	2	3	4	5	9

SHOWCARD IDQ9
IDQ9. How often do you **associate** with others of your ethnic group?

Most of the time	Often	Sometimes	Not often	Almost never	DK/Ref **DO NOT READ OUT**
1	2	3	4	5	9

SHOWCARD IDQ10
IDQ10. How important is it for you to **maintain** your cultural traditions and practices?

Very important	Fairly important	Somewhat important	Not very important	Not important at all	DK/Ref **DO NOT READ OUT**
1	2	3	4	5	9

Preamble for single response: You identified your ethnicity as _____ **[INSERT RESPONSE FROM ETH3]**

Preamble for multiple response: You identified your ethnicity as _____ and _____ (and _____, etc.) **[INSERT RESPONSES FROM ETH3]**

SPEC NOTE; If 2+ responses at ETH3 PULL THROUGH 1ST RESPONSE AND ASK IDQ11 AND IDQ12 AND THEN REPEAT FOR 2ND, 3RD RESPONSE, ETC.

IDQ11. How well are you able to understand the spoken language of that group?

SHOWCARD IDQ11-12

Very well	Well	Fairly well	Not very well	No more than a few words or phrases	DK/Ref **DO NOT READ OUT**
1	2	3	4	5	9

SHOWCARD IDQ11-12

IDQ12. How well are you able to speak the language of that group in day-to-day conversation?

Very well	Well	Fairly well	Not very well	No more than a few words or phrases	DK/Ref **DO NOT READ OUT**
1	2	3	4	5	9

Next questions for all

IDQ13. Do you know the name of the place – that is, the village, island, district or town - that your mother's family comes from?

Yes = 1
No = 2 **GO TO IDQ15**
DK/Ref = 9 **GO TO IDQ15**

IDQ14. Does this place have a special significance for you?

Yes = 1
No = 2
DK/Ref = 9

IDQ15. Do you know the name of the place – that is., the village, island, district or town - that your father's family comes from?

Yes = 1
No = 2 **GO TO IDQ17**
DK/Ref = 9 **GO TO IDQ17**

IDQ16. Does this place have a special significance for you?

Yes = 1
No = 2
DK/Ref = 9

IDQ17. Have you, or will you observe any cultural practices specific to your culture <u>while pregnant</u>?

Yes = 1 **GO TO IDQ18**
No = 2 **GO TO ETH5**
DO NOT READ OUT DK/Ref = 9 **GO TO ETH5**

<u>SHOWCARD IDQ18</u>

IDQ18. Please tell me these cultural practices.
Interviewer Instruction: Multiple Response Code all that Apply

consulting an elder about your child's name = 01
stopped cutting your hair = 02
made arrangements for the burial of the placenta = 03
avoided going to a cemetery = 04
avoided certain types of food = 05
eaten specific foods for maternal good health or good luck = 06
restrict travel (not going out) 1 month after the birth = 07
Other (please specify).= 97
DK/Ref = 99

© Growing Up in New Zealand 2009

SHOWCARD ETH5-6

ETH5. Which ethnic group/groups will your child belong to? *Please indicate using the showcard*
Multi response possible Code all that Apply

New Zealand European	1
Māori	2
Samoan	3
Cook Islands Maori	4
Tongan	5
Niuean	6
Tokelauan	7
Fijian	8
Fijian Indian	9
Other Pacific Peoples	10
Indian	11
Sri Lankan	12
Other Asian	13
Chinese	14
Korean	15
Japanese	16
Filipino	17
Cambodian	18
Vietnamese	19
Other Southeast Asian	20
Australian	21
British and Irish	22
Dutch	23
Greek	24
Polish	25
South Slav (formerly Yugoslav)	26
Italian	27
German	28
Other European	29
Middle Eastern	30
Latin American/Hispanic	31
African	32
New Zealander **NOT ON CARD**	40
Other Ethnicity Please specify	96
Other Ethnicity Please specify	97
DK/Ref	99

People may be treated unfairly for a number of reasons such as a disability, height, weight, gender, or their ethnicity or culture. The following questions are about unfair treatment based on ethnicity.

ETH6. Earlier you identified your ethnicity. These next questions are about reactions to your ethnicity. With which ethnic group, do other people usually classify you in New Zealand?

<u>SHOWCARD ETH5-6</u>

Single Response

New Zealand European	1
Māori	2
Samoan	3
Cook Islands Maori	4
Tongan	5
Niuean	6
Tokelauan	7
Fijian	8
Fijian Indian	9
Other Pacific Peoples	10
Indian	11
Sri Lankan	12
Other Asian	13
Chinese	14
Korean	15
Japanese	16
Filipino	17
Cambodian	18
Vietnamese	19
Other Southeast Asian	20
Australian	21
British and Irish	22
Dutch	23
Greek	24
Polish	25
South Slav (formerly Yugoslav)	26
Italian	27
German	28
Other European	29
Middle Eastern	30
Latin American/Hispanic	31
African	32
New Zealander **NOT ON CARD**	40
Other Ethnicity Please specify	96
Other Ethnicity Please specify	97
DK/Ref	99

SHOWCARD ETH7

ETH7. How often do you think about your ethnicity?

Never = 1
At least once a year = 2
At least once a month = 3
At least once a week = 4
At least once a day = 5
At least once an hour = 6
Constantly = 7
DK/Ref = 9

SHOWCARD ETH8

ETH8. Have you ever felt you have been a victim of an **ethnically** motivated attack – that is, verbal or physical abuse to the person or property in New Zealand?

Multi response

Yes, verbal – within the past 12 months = 1
Yes, verbal – more than 12 months ago = 2
Yes physical – within the past 12 months = 3
Yes physical –more than 12 months ago = 4
No = 5
DK/Ref = 9

SHOWCARD ETH9-14

ETH9. Have you ever felt you have been treated unfairly, e.g., treated differently, kept waiting, by a health professional, e.g., doctor, nurse, dentist etc., **because of your ethnicity** in New Zealand?

Multi response

Yes, within the past 12 months = 1
Yes, more than 12 months ago = 2
No = 3
Not Applicable = 4
DK/Ref = 9

SHOWCARD ETH 9-14

ETH10. Have you ever felt you have been treated unfairly at work or been refused a job **because of your ethnicity** in New Zealand?

Multi response

Yes, within the past 12 months = 1
Yes, more than 12 months ago = 2
No = 3
Not Applicable = 4
DK/ref = 9

© Growing Up in New Zealand 2009

SHOWCARD ETH 9-14

ETH11. Have you ever felt you have been treated unfairly when renting or buying housing **because of your ethnicity** in New Zealand?

Multi response

Yes, within the past 12 months = 1
Yes, more than 12 months ago = 2
No = 3
Not Applicable = 4
DK/Ref = 9

SHOWCARD ETH 9-14

ETH12. Have you ever felt you have been treated unfairly by the police, the justice system (courts), or the corrections department (prison, community service, periodic detention, parole, probation) **because of your ethnicity** in New Zealand?

Multi response

Yes, within the past 12 months = 1
Yes, more than 12 months ago = 2
No = 3
Not Applicable = 4
DK/Ref = 9

SHOWCARD ETH 9-14

ETH13. Have you ever felt you have been treated unfairly when asking for loans, a mortgage, hire purchase or credit cards **because of your ethnicity** in New Zealand?

Multi response

Yes, within the past 12 months = 1
Yes, more than 12 months ago = 2
No = 3
Not Applicable = 4
DK/Ref = 9

SHOWCARD ETH 9-14

ETH14. Have you ever felt you have been treated unfairly when attending a place of learning (e.g. Te Kohanga Reo, pre-school, primary school, secondary school, polytechnic, whare wananga, university) **because of your ethnicity** in New Zealand?

Multi response

Yes, within the past 12 months = 1
Yes, more than 12 months ago = 2
No = 3
Not Applicable = 4
DK/Ref = 9

© Growing Up in New Zealand 2009

Section E: Societal Context

The next questions are about you and your household.
First we would like to know about who owns the house you are currently living in.

HH6. Do you, or anyone else who lives here, own or partly own this dwelling (with or without a mortgage)?

Interviewer Note- If owned by a Trust code as 2

Yes = 1 **GO TO HH9**
No = 2 **GO TO HH7**
DK/Ref = 9 **GO TO HH9**

SHOWCARD HH7
Interviewer Note: If respondent mentions 'Church' code as 1
HH7. If nobody who lives here owns this dwelling, who owns it

Private person, trust or business = 1
Family trust = 2
Local Authority or City Council = 3
Housing New Zealand Corporation = 4
Other state-owned corporation or state owned enterprise, or government department or ministry = 5
DK/Ref = 9

HH8. Do you, or anyone else who lives here pay rent to an owner or to an agent for this house/flat?
Interviewer Note: If respondent indicates they pay rent to another organisation (e.g., church) or individual, code as "Yes".

Yes = 1 **GO TO HH10**
No = 2 **GO TO HH9**
DK/Ref = 9 **GO TO HH9**

HH9. Do you, or anyone else who lives here make mortgage payments for this house/flat?

Yes = 1
No = 2
DK/Ref = 9

HH10. How many bedrooms are there in this dwelling? ___________________________________
Interviewer Instruction: Enter as double digits- range from 00

DK/Ref = 99

OCC1. Are you currently attending, studying or enrolled at school or anywhere else?

Yes = 1 **GO TO OCC2**
No = 2 **GO TO OCC3**
DK/Ref = 9 **GO TO OCC3**

SHOWCARD OCC2

OCC2 Is that……….

Full time (20 hours a week or more) = 1
Part time (less than 20 hours per week) = 2
DK/Ref = 9

SHOWCARD OCC3

OCC3. Thinking about any current paid work, that might include working for pay or profit or income for an hour or more, or working in a family business or family farm, or working in a job or business. In that job, which of these are you?

NOTE: Those currently on leave from paid employment count as being in current paid work. Include "Stay at home parents" as code 6.

A paid employee = 1 **GO TO OCC4**
Self-employed and NOT employing others = 2 **GO TO OCC4**
An employer of other persons in my own business = 3 **GO TO OCC4**
Working in a family business or family farm = 4 **GO TO OCC4**
Unemployed = 5 **GO TO OCC30**
Not currently in paid work and not seeking work =6 **GO TO OCC18**
DK/Ref =9 **GO TO OCC30**

OCC4-13 Paid workers only (OCC3 = 1-4)

I'm now going to ask you some questions about the work that you do. This includes all paid work, and any unpaid work in a family business or on a farm that you may be doing. Include jobs from which you are currently on leave

OCC4. How many jobs do you currently have?
[RANGE: 1-10; 99 =DK/Ref]

OCC5. Including overtime, how many hours a week do you usually work in all your jobs?

Interviewer note: If respondent is working variable hours, ask for an average number of hours worked per week over the past 4 weeks.

If on maternity leave, code 0 and **GO TO OCC7**
IF NECESSARY: Overtime includes both paid or unpaid work.
___ hours

[RANGE: 1-90; >90 code as 90; 99 = DK/Ref]

If OCC5 = 99 **GO TO OCC7**
If OCC5 <30 **GO TO OCC6**
If OCC5 >30 **GO TO OCC7**

SHOWCARD OCC6
OCC6. You have said currently that you usually work fewer than 30 hours per week. What is the main reason for your working part-time hours rather than full-time hours?
SINGLE RESP
I've cut back my hours now that I am pregnant 01
Own illness or disability 02
Caring for children 03
Caring for disabled or elderly relatives (not children) 04
Other personal or family responsibilities 05
Going to school, college, university etc. 06
Could not find full-time work 07
Prefer part-time work 08
Involved in voluntary work 09
Attracted to extra pay attached to part-time / casual work 10
Welfare payments or pension may be affected by working full-time 11
Getting business established 12
My preferred job offers only part-time hours 13
Can't get suitable childcare 14
Other (*please specify)* 98
____
DK/Ref 99

OCC7. In the job that you spend the most time on, what is your occupation?
IF NECESSARY: **Some examples of occupations are primary school teacher, clothing machinist, motel manager, receptionist.**
Interviewer note: Probe clear answer. Probe may include "What kind of business/industry is that in? What do they make or do?" Enter 99 if respondent does not know. Do not leave Field blank

SHOWCARD OCC8
OCC8. Looking at **SHOWCARD OCC8**, which one of the categories would you consider your job to be in?
If respondent does not believe their job falls into any of the categories, select 99 DK/Ref.

Manager = 1
Professional = 2
Technician or Trades Worker = 3
Community or Personal Service Worker = 4
Clerical or Administrative Worker = 5
Sales Worker = 6
Machinery Operator or Driver = 7
Labourer = 8
DK/Ref = 99

OCC8A. What tasks or duties do you spend the most time on?
IF NECESSARY: **Some examples of tasks or duties are running a motel, servicing and repairing cars, answering phones. Enter 99 if respondent does not know. Do not leave Field blank. Interviewer note: Probe clear answer. Probe may include: "What kind of work do you do?"**

____

____

Leave

OCC9. Are you planning to take any leave from employment when this baby is born?

Yes = 1
No = 2 **GO TO OCC11**
DK/Ref = 9 **GO TO OCC11**

OCC10. How long do you anticipate your total leave will be, both paid and unpaid?

Number of weeks = 01 **GO TO OCC10A**
Number of months = 02 **GO TO OCC10A**
Number of years and months = 03 **GO TO OCC10A**
DO NOT READ OUT DK/Ref = 99 **GO TO OCC11**

OCC10A. Interviewer Instruction: Enter as digits as required
[RANGE: weeks 01-51; months 01-24; years and months, years 01-21, months 00-11]

			Number of weeks
OR			Number of months
	Number of years		Number of months

OCC11. How much total leave from employment would you prefer to take?

Number of weeks = 01 **GO TO OCC11A**
Number of months = 02 **GO TO OCC11A**
Number of years and months = 03 **GO TO OCC11A**
DO NOT READ OUT DK/Ref = 99 **GO TO OCC30**

OCC11A. Interviewer Instruction: Enter as digits as required
[RANGE: weeks 01-51; months 01-24; years and months, years 01-21, months 00-11]

			Number of weeks
OR			Number of months
	Number of years		Number of months

SPEC INSTRUCTION: IF OCC 10 = 99- **GO TO OCC30**
Pull responses through (Codes 01, 02, or 03) at OCC10A and (Codes 01, 02, or 03) at OCC11A to read.

You have said, that you are **anticipating you will take**.(Insert response at OCC10A) leave and you would **prefer to take** (Insert response at OCC11A) leave.
So, to clarify your…(Interviewer to select and insert appropriate answer at OCC12)

Code at OCC12 when confirmed by respondent.
OCC12.

Anticipated Total Leave is MORE Than your Preferred Leave =1 **GO TO OCC30**
Anticipated Total Leave is LESS Than Preferred Leave = 2 **GO TO OCC13**
Anticipated Total Leave is the SAME AS Preferred Leave = 3 **GO TO OCC30**
DK/Ref = 9 **GO TO OCC30**

<u>SHOWCARD OCC13</u>

OCC13. Why is that? MULTIPLE RESPONSE Code all Mentions

Financial reasons (e.g. can't afford more time off, household budget considerations, not enough paid leave to cover the preferred length of time) = 1
Government regulations about the length of leave entitlement = 2
Company or employer regulations about the length of leave = 3
Professional or work commitments restrict the amount of time that can be taken = 4
Parenting preferences = 5
Other, please specify = 6
DK/Ref = 9

GO TO OCC30.

OCC18 Unpaid workers only (OCC3 = 6)

OCC18. What are the reasons why you are not currently in paid work? Multi response

I'm looking after children/family members = 01
I quit work now that I am pregnant = 02
Own illness or disability = 03
Going to school, college or university etc = 04
Partner earns enough to support them = 05
No jobs available = 06
Can't find a job that interests = 07
Can't find a job with enough flexibility = 08
Can't get suitable child care = 09
It's not worthwhile with child care costs = 10
Would lose government benefits if worked = 11
Other (please specify) = 12
DK/Ref = 99
None of these = 00

*****NOTE: OCC30-OCC32 SKIPPED IF PRG13=2 OR IF PRG14=0*****

OCC30. Is this your first child?

Yes =1 **GO TO OCC33**
No = 2 **GO TO OCC31**
DK/Ref = 9 **GO TO OCC33**

OCC31. Now, thinking about BEFORE your first child was born, what was your main occupation in the job that you had before your first child was born?
***IF NECESSARY:* Some examples of occupations are primary school teacher, clothing machinist, motel manager, receptionist.**

Interviewer note: Probe clear answer. Probe may include "What kind of business/industry is that in? What do they make or do?" Enter 99 if respondent does not know or 97 if they had no job. Do not leave Field blank

No job = 97 **GO TO OCC33**
DK/Ref = 99 **GO TO OCC33**

SHOWCARD OCC32

OCC32. Looking at Showcard 32, which one of the categories would you consider your main job to have been in?
If respondent does not believe their job falls into any of the categories, select 9 DK.

Manager = 1
Professional = 2
Technician or Trades Worker = 3
Community or Personal Service Worker = 4
Clerical or Administrative Worker = 5
Sales Worker = 6
Machinery Operator or Driver = 7
Labourer = 8
DK/Ref = 9

OCC32A. What tasks or duties did you spend the most time on?
IF NECESSARY: **Some examples of tasks or duties are running a motel, servicing and repairing cars, answering phones.**
Interviewer note: Probe clear answer. Probe may include: "What kind of work do you do?"

____

____

Now thinking about, your intentions to work or return to paid work after this baby is born.

OCC33. Do you expect to start or return to paid work – either full or part-time – at some stage after this child is born?

Yes = 1 **GO TO OCC34**
No = 2 **GO TO ED1**
DK/Ref = 9 **GO TO ED1**

OCC34. How old do you expect your child to be when you start or return to paid work, either full or part-time?

Number of months = 01 **GO TO OCC34A**
Number of years and months = 02 **GO TO OCC34A**
DO NOT READ OUT DK/Ref = 99 **GO TO OCC35**

OCC34A. Interviewer Instruction: Enter digits as required. - If less than 1 month enter as 01
[RANGE: months 01-24; years and months, years 01-21, months 00-11]

			Number of months
	Number of years		Number of months

OCC35. Has it been decided who will look after your child when you start or go back to paid work?

Yes = 1 **GO TO OCC36**
No = 2 **GO TO ED1**
DK/Ref = 9 **GO TO OCC36**

SHOWCARD OCC36
OCC36. What sort of child-care do you intend to use when you start or go back to paid work?
Multi response

Childminder in your home (not family) = 01
Childminder in their home (not family) = 02
Partner = 03
Other Family member in your own home = 04
Other Family member in their home = 05
Early Childhood centre or similar = 06
Looking after the baby while I am working = 07
Other (please specify) = 08
DK/Ref = 99

Education

Help text available

SHOWCARD ED1

ED1. Looking at the showcard, what is your highest completed secondary school qualification?

No secondary school qualifications = 1
NZ School Certificate *or* National Certificate/NCEA level 1 = 2
NZ Sixth Form Certificate *or* National Certificate/NCEA level 2 *or* NZ UE before 1986 = 3
NZ Higher School Certificate *or* NZ University Entrance from NZ Bursary *or* National Certificate/NCEA level 3 = 4
NCEA level 4 = 5
other NZ secondary school qualification - please specify ………………………….. = 6
overseas secondary school qualification = 7
DK/Ref = 9

ED2. Apart from secondary school qualifications, do you have any other completed qualifications, the equivalent of 3 months or more full-time study to complete?

Yes = 1 **GO TO ED3**
No = 2 **GO TO FIN1**
DK/Ref = 99 **GO TO FIN1**

ED3. What is your highest completed qualification?

SHOWCARD ED3

Trade Certificate or National Certificate levels 1 - 4 = 1
Diploma below bachelors level (e.g. teachers or nursing diploma) or National Certificate levels 5 or 6 = 2
Bachelor’s degree = 3
Bachelors degree with honours, or postgraduate diploma = 4
Masters Degree = 5
PhD = 6
other - please specify …………………… = 7
DK/Ref = 9

© Growing Up in New Zealand 2009

Income

We would like to ask some questions about your income, both yours personally and that of your household. You may recognise the questions from previous census forms you have filled out. All the information is confidential and it will help us if it is as accurate as possible.

SHOWCARD FIN1

FIN1. Looking at the showcard, what are all the ways that you personally got income in the last 12 months ending today? You can choose as many as you need.
Please do not count loans, including student loans, because they are not income.

Wages, salary, commissions, bonuses etc paid by an employer = 01
Self-employment or business = 02
Interest, dividends, rent, other investments = 03
Regular payments from ACC or a private work accident insurer = 04
NZ Superannuation or Veterans Pension = 05
Other superannuation pensions, annuities (other than NZ Superannuation, Veterans Pension or War Pension) = 06
Unemployment Benefit = 07
Sickness Benefit = 08
Domestic Purposes Benefit = 09
Invalids Benefit = 10
Student Allowance (including scholarships or stipends) = 11
Other government benefits, government income support payments, War pensions, or paid parental leave = 12
Other sources of income, counting support payments from people who do not live in your household = 13
Child support payments = 14
No source of income during that time = 15 **GO TO FIN6**
DK/Ref = 99

FIN2. In the last 12 months what was your personal total income?

Interviewer Instruction: Write in numbers amount mentioned e.g. 20000
$_____________

DK/Ref = 99 **GO TO FIN5**

SHOWCARD FIN3

FIN3. What period does that cover?
Interviewer please specify if not on coded list.

Weekly before tax = 1
Fortnightly before tax = 2
4 weekly before tax = 3
Calendar monthly before tax = 4
Yearly before tax = 5
Weekly after tax = 6
Fortnightly after tax = 7
4 Weekly after tax = 8
Calendar monthly after tax = 9
Yearly after tax = 10
Other Specify = 97
DK/Ref = 99 **GO TO FIN5**

FIN4. So that's **[CAPI feed through of FIN2] [CAPI feed through of FIN3]**, right?

Yes = 1 **GO TO FIN6**
No = 2 **GO TO FIN2**
Re-enter

SHOWCARD FIN5

Only ask if unable to answer FIN2 and FIN3.
FIN5. In the last 12 months what was your personal total income, before tax or anything else was taken out of it?

Loss = 1
Zero income = 2
$1-$5,000 = 3
$5,001-$10,000 = 4
$10,001-$15,000 = 5
$15,001-$20,000 = 6
$20,001-$25,000 = 7
$25,001-$30,000 = 8
$30,001-$40,000 = 9
$40,001-$50,000 = 10
$50,001-$70,000 = 11
$70,001-$100,000 = 12
$100,001 -$150,000 = 13
$150,001 or more = 14
DK/Ref = 99

SKIP FIN6-FIN10 IF HH2=0

SHOWCARD FIN6

Household income
FIN6. Looking at the showcard, what are all the ways that your household got income in the last 12 months ending today? Please include your personal income when answering this question. You can choose as many as you need.

Wages, salary, commissions, bonuses etc paid by an employer = 01
Self-employment or business = 02
Interest, dividends, rent, other investments = 03
Regular payments from ACC or a private work accident insurer = 04
NZ Superannuation or Veterans Pension = 05
Other superannuation pensions, annuities (other than NZ Superannuation, Veterans Pension or War Pension) = 06
Unemployment Benefit = 07
Sickness Benefit = 08
Domestic Purposes Benefit = 09
Invalids Benefit = 10
Student Allowance (including scholarships or stipends) = 11
Other government benefits, government income support payments, War pensions, or paid parental leave = 12
Other sources of income, counting support payments from people who do not live in your household = 13
Child support payments = 14
No source of income during that time = 15 **GO TO FIN11**
DK/Ref = 99

FIN7. In the last 12 months what was your household's total income? Please include your personal income in this total.
Help text available

Interviewer Instruction: Write in numbers amount mentioned e.g. 20000
$_____________

DK/Ref = 99 **GO TO FIN10**

SHOWCARD FIN8

FIN8. What period does that cover?
Interviewer please specify if not on coded list.

Weekly before tax = 1
Fortnightly before tax = 2
4 weekly before tax = 3
Calendar monthly before tax = 4
Yearly before tax = 5
Weekly after tax = 6
Fortnightly after tax = 7
4 Weekly after tax = 8
Calendar monthly after tax = 9
Yearly after tax = 10
Other Specify = 97
DK/Ref = 99 **GO TO FIN10**

FIN9. So that's **[CAPI feed through of FIN2] [CAPI feed through of FIN3**], right?

Yes = 1 **GO TO FIN11**
No = 2 **GO TO FIN7**
Re-enter

SHOWCARD FIN10

Only ask if unable to answer FIN7 and FIN8.
FIN10. In the last 12 months what was your household's total income, before tax or anything else was taken out of it? Please include your personal income in this total.

Loss = 1
Zero income = 2
$1-$5,000 = 3
$5,001-$10,000 = 4
$10,001-$15,000 = 5
$15,001-$20,000 = 6
$20,001-$25,000 = 7
$25,001-$30,000 = 8
$30,001-$40,000 = 9
$40,001-$50,000 = 10
$50,001-$70,000 = 11
$70,001-$100,000 = 12
$100,001 -$150,000 = 13
$150,001 or more = 14
DK/Ref = 99

FIN11. Does this household income regularly contribute money to individuals, organisations or family not living here? This includes sending money overseas. Do not include door-to-door or street appeals.

Yes = 1
No = 2 **GO TO FIN13**
DK/Ref = 9 **GO TO FIN13**

FIN12. How many individuals are dependent on the income from this household for financial support - including those who live both inside and outside of the household, also include yourself?

Interviewer Instruction: Enter as double digits [RANGE: 00-15 for Children and 01-15 for Adults]

FIN12A. So firstly, the number of children?___________________

FIN12B. And the number of adults? _____________________(including yourself)

DK/Ref = 99

FIN13. Apart from yourself and your partner, *if applicable*, do other people contribute to this household's expenses - including those who live outside of the household if applicable?

Yes = 1
No = 2
DK/Ref = 9

FIN14. Are you aware of the "Working for Families" tax credits?

Yes = 1
No = 2 **GO TO TR1**
DK/Ref = 9 **GO TO TR1**

FIN15. Do you or your partner currently receive "Working for Families" tax credits?

Yes = 1
No = 2
DK/Ref = 9

FIN16. Do you or your partner expect to receive "Working for Families" tax credits after this baby is born?

Yes = 1
No = 2
DK/Ref = 9

Transport

TR1. How many motor vehicles, not counting motorbikes, do the people who live here have available for their use?

READ OUT….DON'T count:
Vehicles that belong to visitors
Vehicles that this household borrows occasionally from another household
Vehicles that can be used ONLY for work
Motorbikes

Print number of motor vehicles **(0-10)** _____
DK/Ref = 99

© Growing Up in New Zealand 2009

SHOWCARD TR2

TR2. Do you have a motor vehicle available for your personal use?
READ OUT….DON'T count:
Vehicles that belong to visitors
Vehicles that this household borrows occasionally from another household
Vehicles that can be used ONLY for work
Motorbikes

Yes, always = 1
Yes, sometimes = 2
No = 3
Do not drive = 4
DK/Ref = 9

We would like to ask some questions about the types of transport that you use on a regular basis in your everyday life. For example, when you are travelling to shops, work, friends and other activities.

TR3A. Thinking of the last 7 days, please indicate how you travelled about most regularly. If the last 7 days was clearly unusual, please use a typical 7 days.

SHOWCARD TR3

TR3B. And what is the main form of transport that you use? If you use more than one method of transport in your main type of transport, such as bus then walk, select the one that you use for the longest distances. For example: for a 5km bus trip followed by a 1km walk, pick "Public transport".

Types of transport used regularly last week	TR3A. All types used	TR3B. Main type used
1. Driving yourself in a private car	□	○
2. Driving yourself in a company car	□	○
3. Lifts from family & friends	□	○
4. Motorcycle / scooter	□	○
5. Public transport (bus/train/ferry)	□	○
6. Cycle	□	○
7. Walk	□	○
8. Taxi	□	○
9. Other, Please specify	□	○
99. DK/Ref	□	○

IF ONLY ONE CODE AT TR3A, CODE TR3B AS NUMERIC CODE CHOSEN AT TR3A AND GO TO NE1

Section F: Neighbourhood & Environment

Now I'd like to ask you about the local neighbourhood in which you live. 'Neighbourhood' usually means the immediate area that you live in, approximately within a 15 minute walk of your house if you live in city, town or village. If you live in a rural area we mean the area around your house where you know people as 'neighbours'.

NE1. How many times have you moved house <u>in the past five years</u>? _________________
Interviewer Instruction: Enter as double digits [RANGE: 1-20, code >20 as 20]

Not moved in past 5 years / never moved = 97
DK/Ref = 99

NE2. How long have you lived in this *neighbourhood*. Include the time living in another house if it was still in the same neighbourhood?
SPEC CHECK answer at BG2 (Use as upper range limit for number of years)

Number of months = 01 **GO TO NE2A**
Number of years and months = 02 **GO TO NE2A**
DO NOT READ OUT DK/Ref = 99 **GO TO NE3**

NE2A. **Interviewer Instruction: Enter digits as required, If less than 1 month code as 01 Months**

[RANGE: months 01-24; years and months, years 01-50; months 00-11]

			Number of months
	Number of years		Number of months

NE3. From today, how long do you intend to stay in this neighbourhood?

Number of months = 01 **GO TO NE3A**
Number of years and months = 02 **GO TO NE3A**
DO NOT READ OUT DK/Ref = 99 **GO TO NE4**

NE3A. **Interviewer Instruction: Enter digits as required, If less than 1 month code as 01 Months**
If >50 Years or respondent says "forever", code 50 years 00 Months.

[RANGE: months 01-24; years and months, years 01-50; months 00-11].

			Number of months
	Number of years		Number of months

© Growing Up in New Zealand 2009

We are interested in why families come to live in different neighbourhoods. The next question asks about the important factors that led to you living where you are now.

SHOWCARD NE4

NE4. Why do you live in this neighbourhood? Please mention all that apply
Interviewer note: If respondent says they bought or rented a house, ask why they chose this neighbourhood and code this response

For Work = 01
Good Education = 02
Friends/Family Nearby = 03
Better or More Affordable Housing/Rental = 04
With Similar Population Groups = 05
Good and Safe Neighbourhood = 06
Handy to Shops and Other Amenities = 07
Pregnancy Related Reason = 08
I like the local lifestyle = 09
My spouse/partner/family have a house here = 10
Other (please specify)_______ = 97
DK/Ref = 99

Thinking about your local neighbourhood and your experiences, could you please indicate how much you agree or disagree with the following statements:

SHOWCARD NE5-15

NE5. I would be sorry if I had to move away from the people in my neighbourhood.

Strongly agree	Agree	Neither agree nor disagree	Disagree	Strongly disagree	DK/Ref **DO NOT READ OUT**
5	4	3	2	1	9

NE6. I have a lot in common with people in my neighbourhood**.**

Strongly agree	Agree	Neither agree nor disagree	Disagree	Strongly disagree	DK/Ref **DO NOT READ OUT**
5	4	3	2	1	9

NE7. My neighbours treat me with respect.

Strongly agree	Agree	Neither agree nor disagree	Disagree	Strongly disagree	DK/Ref **DO NOT READ OUT**
5	4	3	2	1	9

NE8. I like living where I live.

Strongly agree	Agree	Neither agree nor disagree	Disagree	Strongly disagree	DK/Ref **DO NOT READ OUT**
5	4	3	2	1	9

NE9. I am good friends with some people in the neighbourhood.

Strongly agree	Agree	Neither agree nor disagree	Disagree	Strongly disagree	DK/Ref **DO NOT READ OUT**
5	4	3	2	1	9

NE10. I generally trust my neighbours to look out for my property.

Strongly agree	Agree	Neither agree nor disagree	Disagree	Strongly disagree	DK/Ref **DO NOT READ OUT**
5	4	3	2	1	9

NE11. If I no longer lived here, hardly anyone around here would notice.

Interviewer note: "Hardly anyone around here" refers to people in the neighbourhood.

Strongly agree	Agree	Neither agree nor disagree	Disagree	Strongly disagree	DK/Ref **DO NOT READ OUT**
5	4	3	2	1	9

NE12. I have little to do with people in this neighbourhood.

Strongly agree	Agree	Neither agree nor disagree	Disagree	Strongly disagree	DK/Ref **DO NOT READ OUT**
5	4	3	2	1	9

NE13. It is safe to walk around the neighbourhood at night.

Strongly agree	Agree	Neither agree nor disagree	Disagree	Strongly disagree	DK/Ref **DO NOT READ OUT**
5	4	3	2	1	9

NE14. Children are safe walking around the neighbourhood during the day.

Strongly agree	Agree	Neither agree nor disagree	Disagree	Strongly disagree	DK/Ref **DO NOT READ OUT**
5	4	3	2	1	9

© Growing Up in New Zealand 2009

SHOWCARD NE15

NE15. How much influence do you think the people in your neighbourhood can have in making the neighbourhood or local community a better place to live?

No impact	Small impact	Moderate impact	Large impact	DK/Ref **DO NOT READ OUT**
4	3	2	1	9

SHOWCARD NE16

NE16. What would make this neighbourhood or local community a better place for you? Please mention all that apply

I am happy as it is = 01
More policing to make it safer = 02
Less rubbish lying around = 03
More neighbourhood get togethers / community events = 04
Community/neighbourhood watch = 05
More opportunities for jobs = 06
No violence and crime = 07
Better access to public transport = 08
More affordable housing = 09
Community places for getting together = 10
More recreational areas = 11
Safer road and footpaths = 12
A restaurant/café = 13
Lower noise pollution = 14
More neighbourliness (being friendly, looking out for and respecting each other) = 15
Better shops and amenities = 16
Other (please specify) = 97
DK/Ref = 99

Community belonging
Now we will be talking about Community or communities. When we ask about your '*local community*' it usually means a wider area than your neighbourhood, but 'community' can also mean groups of people that you have things in common with.

NE17. Some people feel they belong to a community because of things like family ties, a school, where they live or maybe a church or club. Do you feel you belong to any communities <u>at the moment</u>?

Yes = 1
No = 2 **GO TO HD1**
DK/Ref = 9 **GO TO HD1**

SHOWCARD NE18

NE18. What is the community or communities of people to which you belong based around? Please mention all that apply

Interests groups = 01
Religion = 02
Whanau = 03
Neighbourhood = 04
Schools = 05
Internet based groups (e.g. social network sites) = 06
Children's sport or activity groups = 07
Adult sport groups = 08
Work or professional groups = 09
Marae = 10
Antenatal or baby support groups = 11
Cultural groups = 12
Other (please specify)_______ = 97
DK/Ref = 99

HD1. Finally, to finish this questionnaire we would like to ask you about the hopes, dreams and expectations you have for your baby. Please give us one or two sentences about the hopes, dreams and expectations you have for your baby.

Enter 99 if respondent does not know. Do not leave Field blank
DO NOT PROBE- ENTER VERBATIM ONLY-NO CLARIFICATION REQUIRED
